# *Globisporangium* and *Pythium* Species Associated with Yield Decline of Pyrethrum (*Tanacetum cinerariifolium*) in Australia

**DOI:** 10.3390/plants12061361

**Published:** 2023-03-17

**Authors:** Yuzhu Liu, Niloofar Vaghefi, Peter K. Ades, Alexander Idnurm, Aabroo Ahmed, Paul W. J. Taylor

**Affiliations:** 1 School of Agriculture and Food, Faculty of Science, University of Melbourne, Parkville, VIC 3010, Australia; 2 School of Ecosystem and Forest Sciences, Faculty of Science, University of Melbourne, Parkville, VIC 3010, Australia; 3 School of BioSciences, Faculty of Science, University of Melbourne, Parkville, VIC 3010, Australia; 4 Department of Plant Sciences, University of Saskatchewan, Saskatoon, SK S7N2R6, Canada

**Keywords:** oomycetes, soil-borne pathogens, yield decline, pyrethrum

## Abstract

Pyrethrum (*Tanacetum cinerariifolium*) cultivation in Australia, which accounts for the majority of global production of natural insecticidal pyrethrins, is affected by a persistent yield decline which in part is caused by a complex of pathogens. *Globisporangium* and *Pythium* species were isolated from crown and roots of pyrethrum plants showing stunting and brown discoloration of crown tissue, and from soil adjacent to diseased plants from yield-decline-affected sites in Tasmania and Victoria, Australia. Ten known *Globisporangium* species (*Globisporangium attrantheridium*, *G. erinaceum*, *G. intermedium*, *G. irregulare*, *G. macrosporum*, *G. recalcitrans*, *G. rostratifingens*, *G. sylvaticum, G. terrestris* and *G. ultimum* var. *ultimum*), two new *Globisporangium* species (*Globisporangium capense* sp. nov. and *Globisporangium commune* sp. nov.) and three *Pythium* species (*Pythium diclinum/lutarium*, *P. tracheiphilum* and *P. vanterpoolii*) were identified through morphological studies and multigene phylogenetic analyses using ITS and *Cox1* sequences. *Globisporangium ultimum* var. *ultimum, G. sylvaticum, G. commune* sp. nov. and *G. irregulare* were most abundant. *Globisporangium attrantheridium, G. macrosporum* and *G. terrestris* were reported for the first time in Australia. Seven *Globisporangium* species were pathogenic on both pyrethrum seeds (in vitro assays) and seedlings (glasshouse bioassays), while two *Globisporangium* species and three *Pythium* species only caused significant symptoms on pyrethrum seeds. *Globisporangium irregulare* and *G. ultimum* var. *ultimum* were the most aggressive species, causing pyrethrum seed rot, seedling damping-off and significant plant biomass reduction. This is the first report of *Globisporangium* and *Pythium* species causing disease in pyrethrum globally and suggests that oomycete species in the family *Pythiaceae* may have an important role in the yield decline of pyrethrum in Australia.

## 1. Introduction

*Tanacetum cinerariifolium* (Trevir.) Sch. Bip., known as pyrethrum, is an herbaceous perennial plant, commercially grown for extraction of natural insecticidal pyrethrins from the oil glands of the outer surface of the achene [1]. In Australia, pyrethrum is grown in the northwest of Tasmania and the Ballarat region of Victoria. Seeds are directly sown in late winter and early spring. Field-dried flower heads are harvested 15–18 months after sowing. After first harvest, pyrethrum will regrow and may be harvested annually in summer for four to eight years while maintaining persistent yield [2,3]. However, over the past 15 years, there has been a lack of persistence within the crop cycle, resulting in a decline of pyrethrum regrowth, and hence reduced yield [3]. As a consequence, most pyrethrum fields in Australia are currently terminated after the first or second harvest.

Typical symptoms of yield-decline-affected pyrethrum plants include stunting, reduced root growth, brown discoloration of crown tissue and leaf lesions, which results in poor regrowth in the following seasons after the first harvest [4]. While the cause of pyrethrum yield decline is not known, soil-borne fungal pathogens have been readily isolated from symptomatic crown and root tissues of yield-decline-affected pyrethrum plants and shown to reduce plant growth and biomass. Pethybridge et al., 2008 reported that *Sclerotinia minor* and *S. sclerotiorum* caused pyrethrum crown and root rot [5]. *Paraphoma vinacea* was also shown to cause dull-tan to reddish-brown discoloration of crown tissue, which resulted in significant reduction of pyrethrum biomass [6]. The foliar pathogen that causes pyrethrum ray blight, *Stagonosporopsis tanaceti*, was also shown to infect pyrethrum crown tissue, resulting in reduced plant growth [7]. Moslemi, Ades, Groom, Nicolas and Taylor [4] isolated *Fusarium oxysporum* and *F. avenaceum* from symptomatic pyrethrum crown and root tissues and showed that these pathogens significantly impacted pyrethrum plant growth. However, until now, all efforts have been focused on the identification of fungal pathogens, while oomycete pathogens such as *Pythium* and *Phytophthora* have been neglected.

Plant diseases caused by *Pythium*-like organisms are widespread in Australia. *Pythium* soft rot (PSR) of ginger occurs in most ginger growing regions of the world [8]. A combination of nine *Pythium*-like species has been reported as causal agents of PSR in Queensland, Australia [8]. Petkowski et al. [9] reported that a *Pythium* disease complex consisting of 11 *Pythium*-like species contributed to root rot of parsnip and parsley in south-eastern Australia. Callaghan et al. [10] reported that 11 *Pythium*-like species were associated with yield loss in processing tomatoes in Victoria, Australia, with *Pythium aphanidermatum*, *Globisporangium ultimum* and *Globisporangium irregulare* identified as the most aggressive species, causing severe damage or death on seeds, seedlings and plants at later stage of plant growth. Globally, *Pythium*-like species were also recorded as pathogenic on *Asteraceae* plants. In Japan, five *Pythium*-like species including *Globisporangium ultimum* var. *ultimum*, *Pythium dissotocum*, *Globisporangium sylvaticum*, *Phytopythium oedochilum* and *Phytopythium helicoides*, caused severe root and stem rot of chrysanthemum [11]. In North Carolina, *G. irregulare* and *P. aphanidermatum* were reported pathogenic on Gerbera daisy [12]. However, there have not been any reports of *Pythium* species causing damping off or affecting the establishment of seedlings and subsequent growth of pyrethrum plants globally.

The genus *Pythium* Pringsheim, nom. cons., sensu lato (s.l.), non *Pythium* Nees was originally described by Pringsheim [13]. Initially, species of *Pythium* s.l. were identified by comparing morphological characteristics using the taxonomic keys primarily developed by Van der Plaats-Niterink [14]; however, these keys are not discriminative enough to differentiate closely related species [15,16]. Multilocus phylogenetic analysis based on multiple gene sequences is now the preferred approach to overcome the limitations of morphological characterization in fungi and pseudofungi, including oomycetes. The Internal Transcribed Spacers and the intervening 5.8S region (ITS) of nuclear ribosomal DNA (nrDNA) has been commonly applied for rapid and accurate identification of *Pythium* spp. to species levels. Nonetheless, for some species, ITS sequences show low divergence among phylogenetically closely related species or intraspecific variation within species [16]. Thus, a combination of morphological characters and sequences of the ITS region and cytochrome *c* oxidase subunit I (*Cox1*) [17] of the mitochondrial genome has been commonly used to identify *Pythium* spp.

The classification of *Pythium* has long been controversial, even when using molecular phylogeny. Lévesque and De Cock [15] divided 116 species and varieties of *Pythium* s.l. into 11 major phylogenetic clades (A to K) based on sequences of ITS and D1-D3 regions of the large subunit (LSU) of nrDNA. Species in Clade K were reclassified as the genus *Phytopythium*, which is an intermediate group placed between *Pythium* and *Phytophthora* according to later phylogenetic analyses based on ITS and *Cox2* [18,19]. This classification was further supported by De Cock et al. [20] using sequences of LSU and the small subunit (SSU) of nrDNA and *Cox1*. A phylogenetic analysis of the genus *Pythium* based on sequences of D1/D2 regions of LSU and *Cox2* showed that *Pythium* s.l. could be classified into five genera, including *Ovatisporangium* (*Phytopythium*)*, Globisporangium, Elongisporangium, Pilasporangium* and *Pythium* sensu stricto (s.s.). This splitting of *Pythium* s.l. was further confirmed by whole-genome sequencing and phylogenomic analysis [21]. In this study, we use *Pythium*-like species to refer to species of these five genera.

The aims of this study were to (1) isolate *Pythium*-like species from symptomatic pyrethrum root and crown tissue, and from soil adjacent to symptomatic plants growing in yield-decline affected fields; (2) identify *Pythium*-like species using multigene phylogenetics and morphological characterization; and (3) assess the pathogenicity of the identified *Pythium*-like species.

## 2. Results

### 2.1. Pythium-like Species Identified from Pyrethrum Fields

A total of 62 isolates of *Pythium* and *Globisporangium* species were obtained from either pyrethrum plant tissues or the surrounding soil collected from the 16 sampled sites (Table 1 and Table 2). Although apple and rose baiting and PAR medium are multipurpose selective media for isolating *Phytophthora* and *Pythium*-like species from plant tissue and soil [22,23,24,25], no *Phytophthora* species were isolates from the monitored sites. A total of 15 *Pythium* and *Globisporangium* species, including 10 known *Globisporangium* species, 2 new *Globisporangium* species and 3 *Pythium* species, were initially identified by morphological characteristics [14] and based on BLAST searches with their ITS sequences, and were confirmed further by phylogenetic analysis (results presented in Section 2.2). However, the isolates VPRI 43974, VPRI 43975 and VPRI 43984 were still not distinguished between *P. diclinum* and *P. lutarium*; hence, they are referred to as *P. diclinum*/*lutarium.* Cultures of the isolates were deposited at the Victorian Plant Pathogen Herbarium (VPRI), Victoria, Australia, or in the Westerdijk Fungal Biodiversity Institute (CBS), Utrecht, The Netherlands.

*Pythium tracheiphilum* and *G. intermedium* were recovered most frequently from pyrethrum crown tissues, followed by *G. irregulare*, *G. rostratifingens* and *P. vanterpoolii*. Eleven *Pythium* and *Globisporangium* species were isolated from pyrethrum root tissues, and the most frequent species were *G. commune* sp. nov. and *G. sylvaticum.* Seven *Pythium* and *Globisporangium* species were isolated from soil. *Globisporangium ultimum* var. *ultimum* and *G. sylvaticum* were most frequent overall. However, *G. ultimum* var. *ultimum* was only isolated from soil and was most frequently isolated across seven sites. *Globisporangium irregulare*, *G. commune* sp. nov. and *G. sylvaticum* were isolated from four sites. The *Pythium*-like isolates recovered from plant tissues were mostly isolated from pyrethrum plants showing stunting and brown discoloration of crown tissue in winter rather than healthy plants. Only three isolates—*G. erinaceum*, *G. irregulare* and *G. attrantheridium*—were isolated from healthy mature plants, and only one isolate of *G. rostratifingens* was isolated from healthy pyrethrum seedlings, while the rest of the species were isolated from symptomatic plants.

The majority of *Pythium* and *Globisporangium* isolates (75.8%) were isolated from pyrethrum fields in winter (Table 2). Sampling of six sites in Tasmania in June 2020 had the highest number of *Pythium* and *Globisporangium* isolates and species. Sampling in December 2020 and November 2021 was carried out at the same six sites in Victoria. All sites were overall healthy and uniform in both samplings. However, in the latter sampling, a few plants showed wilting and stunting. Moreover, internal discoloration and necrosis of the crown tissue were observed in some plants (15-months-old) with healthy appearance, and more *Pythium* and *Globisporangium* isolates were recovered from these plants.

### 2.2. Phylogenetic Analysis

#### 2.2.1. ITS Trees

**Figure 1:**
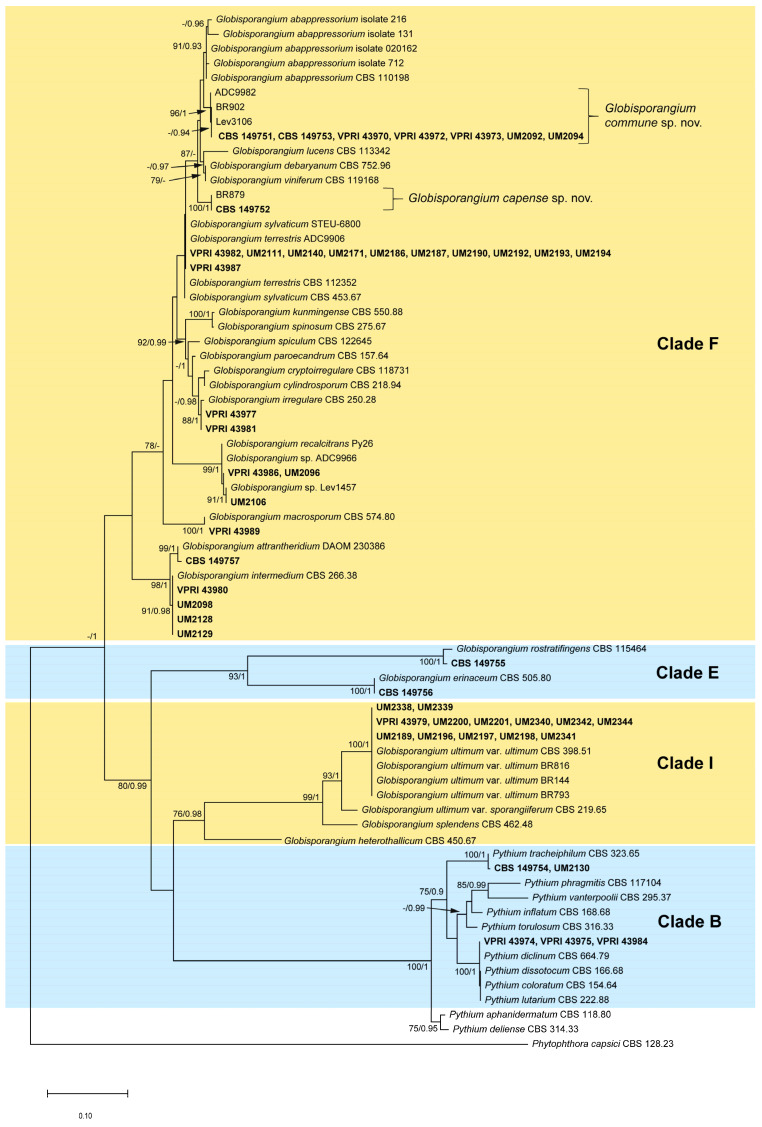
Maximum Likelihood (ML) phylogeny showing relationships among *Pythium*-like species based on ITS sequences. Isolates obtained and sequenced in this study are shown in bold. The ML bootstrap support values greater than 75% and Bayesian posterior probability values greater than 0.90 are indicated at internodes. The scale bar shows the number of nucleotide changes per site. The analysis involved 71 nucleotide sequences. The tree was rooted with *Phytophthora capsici* CBS 128.23 [26]. The clades highlighted were decided based on established phylogeny from Lévesque and De Cock [15].

The alignment of 71 ITS sequences was 877 bp long. The Bayesian inference (BI) analysis lasted for 775,000 generations, resulting in 15,502 trees in total, and the consensus tree and posterior probabilities (PP) were calculated from 11,625 trees. For Maximum Likelihood (ML) analysis, the tree with the highest log likelihood (−5884.28) is shown. The topologies obtained through ML and BI analyses were similar; thus, the BI posterior probabilities (PP) were plotted on the ML tree (Figure 1). *Pythium* and *Globisporangium* isolates collected from pyrethrum clustered within Clade B, Clade F, Clade E and Clade I of 11 *Pythium* clades described by Lévesque and De Cock [15]. Twenty-eight of these pyrethrum isolates clustered with type-derived strains or strains used by Van der Plaats-Niterink [14] for descriptions of *G. attrantheridium*, *G. erinaceum*, *G. intermedium, G. irregulare*, *G. macrosporum*, *G. recalcitrans*, *G. rostratifingens*, *G. ultimum* var. *ultimum* and *P. tracheiphilum* with high bootstrap support values (ML = 84–100%) and high posterior probabilities values (PP = 0.99–1). Isolates CBS 149751, CBS 149753, VPRI 43970, VPRI 43972, VPRI 43973, UM2092, UM2094 and CBS 149752 were nested within Clade F but did not cluster with any known species. Isolates BR879, BR902, Lev 3106 and ADC 9982, which were identified as putative new *Pythium* species by Robideau, De Cock, Coffey, Voglmayr, Brouwer, Bala, Chitty, Désaulniers, Eggertson and Gachon [17], showed 99 to 100% sequence identity to these eight unknown isolates based on the results of ITS and *Cox1* sequences BLAST. Isolate CBS 149752 clustered with isolate BR879 with maximum bootstrap support and posterior probabilities, while CBS 149751, CBS 149753, VPRI 43970, VPRI 43972, VPRI 43973, UM2092 and UM2094 formed a monophyletic clade with isolates BR902, Lev3106 and ADC9982 with maximum bootstrap support and posterior probabilities. Isolates VPRI 43974, VPRI 43975 and VPRI 43984 were placed in a well-supported subclade with type strains of *P. diclinum*, *P. dissotocum*, *P. coloratum* and *P. lutarium* but could not be identified at species level. Isolates VPRI 43982, UM2111, UM2140, UM2171, UM2186, UM2187, UM2190, UM2192, UM2193, UM2194 and VPRI 43987 clustered with *G. sylvaticum* and *G. terrestris* but with low support (ML bootstraps < 75%, BI PP < 90%).

#### 2.2.2. Two-Loci Trees

**Figure 2:**
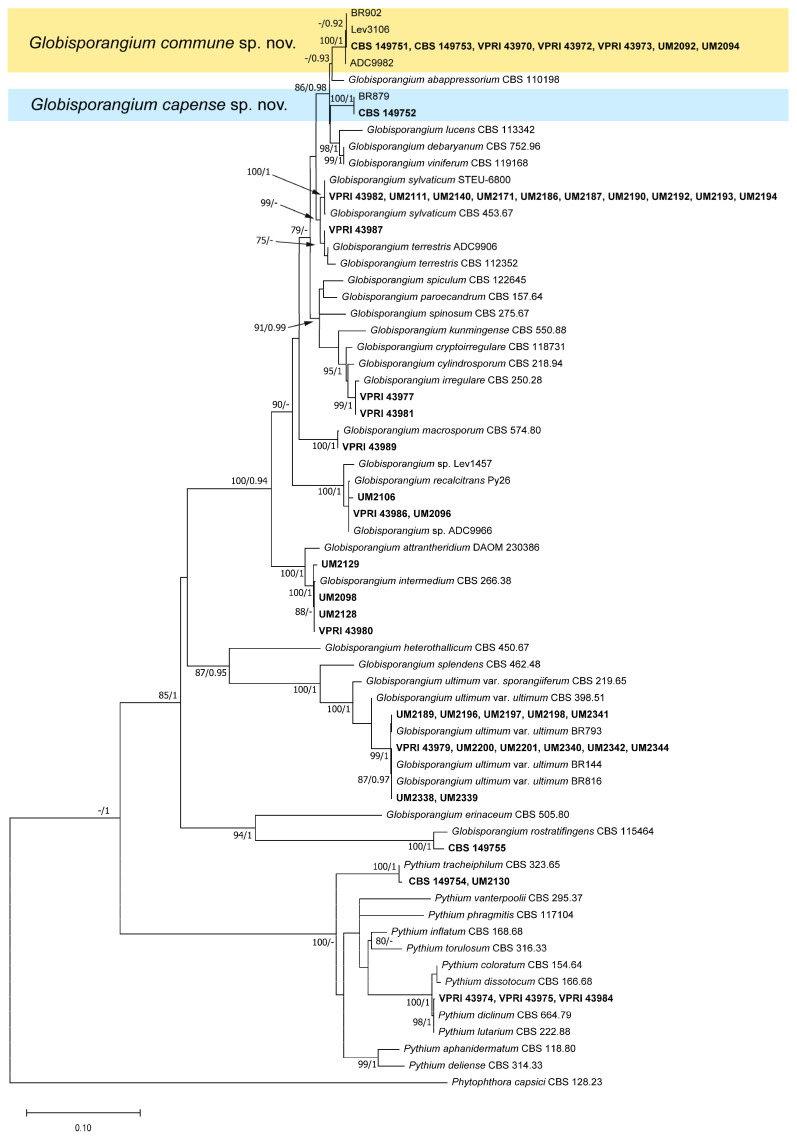
Maximum Likelihood (ML) phylogeny showing relationships among *Pythium*-like species based on concatenated ITS and *Cox1* sequences. Isolates obtained and sequenced in this study are shown in bold. The ML bootstrap support values greater than 75% and Bayesian posterior probability values greater than 0.90 are indicated at internodes. The scale bar shows the number of nucleotide changes per site. The analysis involved 65 nucleotide sequences. The tree was rooted with *Phytophthora capsici* CBS 128.23 [26]. Two new species are highlighted in color.

To determine *Pythium* and *Globisporangium* species identification for isolates which were not identified by ITS sequence alone, *Cox1* sequences were added to the phylogenetic analysis. Based on the concordant results of ITS or *Cox1* trees (Figure 2 and Supplementary Figure S1), BI and ML phylogenetic trees were constructed using a concatenated alignment of ITS and *Cox1* sequences (total length: 1415 bp, ITS: 873 bp and *Cox1*: 542 bp). The Bayesian analysis lasted for 910,000 generations, resulting in 18,202 trees in total, and the consensus tree and posterior probabilities (PP) were calculated from 13,652 trees. For Maximum Likelihood (ML) analysis, the tree with the highest log likelihood (−9516.36) is shown. The topologies obtained through ML and BI analyses were similar; thus, the BI posterior probabilities were plotted on the ML tree (Figure 2). Similar to the results shown in the ITS tree, isolates CBS 149751, CBS 149753, VPRI 43970, VPRI 43972, VPRI 43973, UM2092, UM2094 and CBS 149752 formed two monophyletic clades with maximum bootstrap support and posterior probabilities. Isolates VPRI 43982, UM2111, UM2140, UM2171, UM2186, UM2187, UM2190, UM2192, UM2193 and UM2194 clustered with the type strain of *G. sylvaticum* with maximum support, while VPRI 43987 clustered with the type strain *G. terrestris* with ML bootstrap value of 75%. Isolates VPRI 43974, VPRI 43975 and VPRI 43984 were still not distinguished between *P. diclinum* and *P. lutarium*; hence, they are referred to as *P. diclinum*/*lutarium.*

The two pyrethrum isolates of *P. vanterpoolii* were excluded because their ITS sequences were not available. However, they were included in the *Cox1* region tree (Supplementary Figure S1).

### 2.3. Taxonomy

***Globisporangium capense*** Y. Liu, N. Vaghefi and P.W.J. Taylor, sp. nov.

MycoBank: MB847203

*Etymology:* Named after Table Cape, the prominent local landmark in the Wynyard region.

*Holotype:* CBS 149752, a culture derived from root tissue of diseased *Tanacetum cinerariifolium*, Wynyard, Tasmania, Australia, in June 2020, by K. Groom, stored in a metabolically inactive state in the Westerdijk Fungal Biodiversity Institute (CBS), Utrecht, The Netherlands.

**Figure 3:**
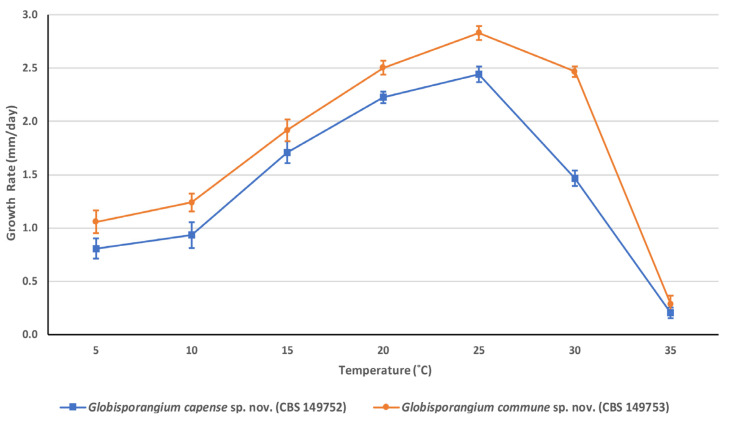
Average radial growth rate of *Globisporangium capense* sp. nov. and *Globisporangium commune* sp. nov. per day on PCA at different temperatures. Error bars represent the mean ± standard deviation.

Colony characteristics—The oomycete grows well on CMA, PCA and PDA. Colonies on these media are fluffy without a specific pattern. Hyphae hyaline are up to 8.3 µm wide and well branched. Average growth rates on PCA 8.1 mm day^−1^ at 5 °C, 9.3 mm day^−1^ at 10 °C, 17.1 mm day^−1^ at 15 °C, 22.3 mm day^−1^ at 20 °C, 24.4 mm day^−1^ at 25 °C, 14.7 mm day^−1^ at 30 °C and 2.0 mm day^−1^ at 35 °C. Cardinal temperatures: optimum 25 °C, minimum 0.5 °C and maximum 35.5 °C (Figure 3).

**Figure 4:**
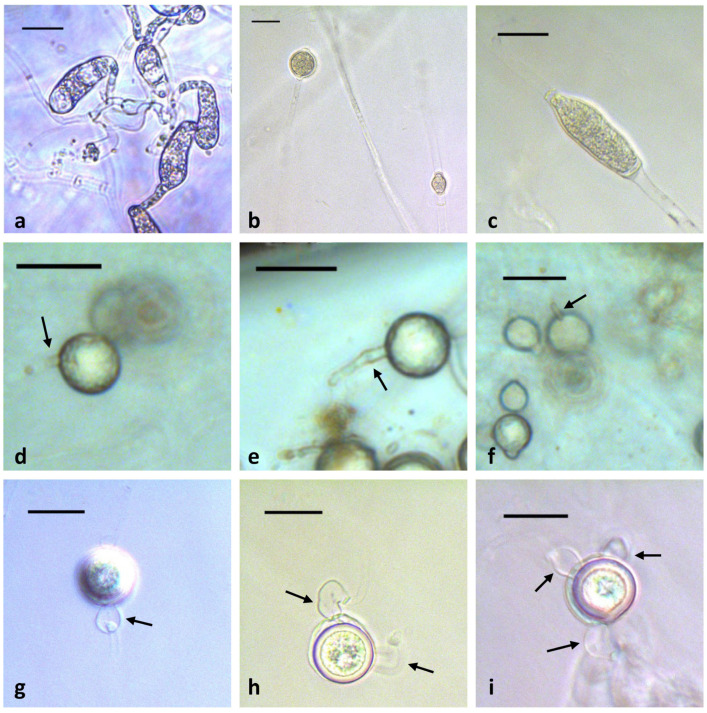
Morphological structures of *Globisporangium capense* sp. nov. (**a**) Clavate appressoria in chain; (**b**) young globose and doliiform intercalary sporangia; (**c**) young elongate intercalary sporangia; (**d**–**f**) sporangia with discharge tubes (arrows); (**g**) plerotic oospore with a hypogynous antheridium (arrow); (**h**) aplerotic oospore with two crook-necked antheridia (arrows); (**i**) aplerotic oospore with three antheridia (arrows). Scale bar: 20 µm.

*Appressoria*: sickle-shaped, sausage-shaped or clavate, often catenulate, abundant on CMA, up to 66.3 µm long and 20.8 µm wide (Figure 4a). *Sporangia*: mainly globose, sometimes subglobose, doliiform, limoniform, elongate or irregular, 9.9–22.4 µm diam (av. 16.5 µm), terminal or intercalary (Figure 4b–f). *Vesicles* and *zoospores*: formed on sterile rye leaves on aqueous cultures after 14 days at 20 °C. Discharge tubes up to 39.9 µm long. Some sporangia do not develop zoospores but may germinate directly by one or more hyphae. *Oogonia*: produced in single culture, globose, smooth, 12.9–23.9 µm in diameter (av. 19.7 µm), terminal or intercalary, sometimes terminal on lateral branches (Figure 4g–i). Oogonial stalks straight. *Antheridia*: often 1–3 per oogonium, globose, or sac-shaped, or clavate, crook-necked, apical or sometimes lateral contact with oogonia, hypogynous, monoclinous, monoclinous sessile or diclinous (Figure 4g–i). Antheridial stalks unbranched. *Oospores*: mostly plerotic, sometimes aplerotic, globose, 11.4–20.9 µm in diameter (av. 17.0 µm), single in an oogonium, colorless with a wall 1.0–3.54 µm thick (av. 2 µm) (Figure 4g–i).

Known geographic distribution: Australia and Canada.

Notes: *Globisporangium capense* belongs to *Globisporangium* genus *sensu* Uzuhashi et al. [27], and belongs to clade F *sensu* Lévesque and De Cock [15], related to *Globisporangium abappressorium* (Paulitz and M. Mazzola) Uzuhashi, Tojo and Kakish. This species is differentiated from other species of clade F by the unique sequences of nuclear ribosomal RNA and mitochondrial genes.

***Globisporangium commune*** Y. Liu, N. Vaghefi and P.W.J. Taylor, sp. nov.

MycoBank: MB847204

*Etymology*: Named based on the fact that the isolates were collected from multiple sites over large geographical distances of pyrethrum production.

*Holotype*: CBS 149753, a culture derived from root tissue of diseased *Tanacetum cinerariifolium*, Wynyard, Tasmania, Australia, in June 2020, by K. Groom, stored in a metabolically inactive state in the Westerdijk Fungal Biodiversity Institute (CBS), Utrecht, The Netherlands.

Colony characteristics—The oomycete grows well on CMA, PCA and PDA. Colonies on these media are fluffy without a specific pattern. Mean hyphae are hyaline, up to 10.2 µm wide and well branched. Average growth rates on PCA are 10.6 mm day^−1^ at 5 °C, 12.4 mm day^−1^ at 10 °C, 19.2 mm day^−1^ at 15 °C, 25.0 mm day^−1^ at 20 °C, 28.3 mm day^−1^ at 25 °C, 24.7 mm day^−1^ at 30 °C and 2.9 mm day^−1^ at 35 °C. Cardinal temperatures: optimum 25 °C, minimum 0.5 °C and maximum 35.5 °C (Figure 3).

**Figure 5:**
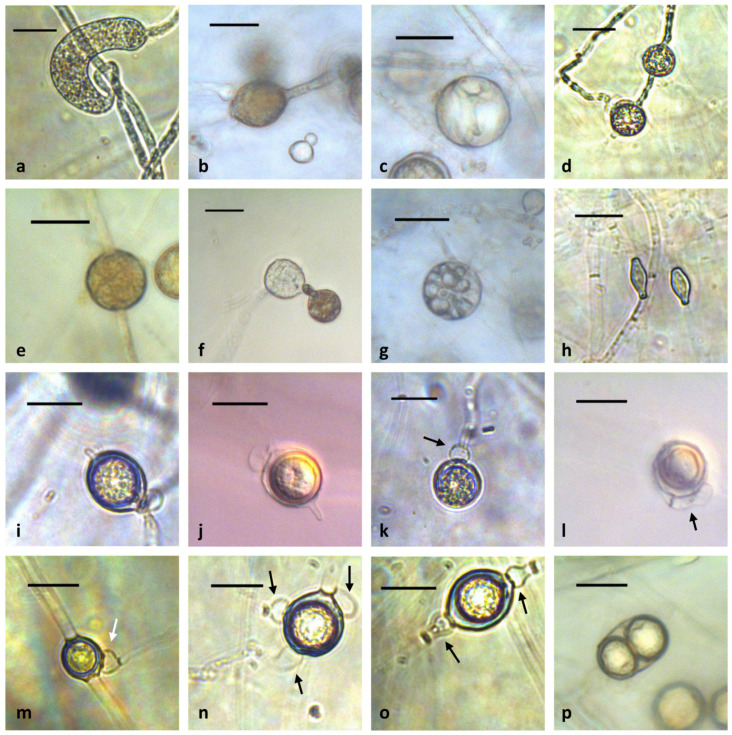
Morphological structures of *Globisporangium commune* sp. nov. (**a**) sausage-shaped appressorium; (**b**) lemon-shaped sporangium; (**c**) empty sporangium; (**d**) catenulate globose sporangia; (**e**) intercalary globose sporangium; (**f**) early stage of vesicle formation; (**g**) zoospores in vesicle; (**h**) doliiform hyphal swellings; (**i**,**j**) oogonium that contains aplerotic oospore with a papilla; (**k**) plerotic oospore with a hypogynous antheridium (arrow); (**l**) elongated antheridial cell (arrow) that makes broad contact with an oogonium; (**m**) intercalary oogonium with a diclinous antheridium (arrow); (**n**) aplerotic oospore with three antheridia (arrows); (**o**) intercalary oogonium with two monoclinous antheridia (arrows); (**p**) two oospores in an oogonium. Scale bar: 20 µm.

*Appressoria*: sausage-shaped, sickle-shaped or clavate, often present, up to 70.5 µm long and 19.2 µm wide (Figure 5a). *Sporangia*: globose, rarely lemon-shaped, obovoid or ellipsoid, 13.0–27.9 µm in diameter (av. 20.6 µm), terminal or intercalary, occasionally catenulate (Figure 5b–g). *Vesicles* and *zoospores*: formed on sterile rye leaves on aqueous cultures after 14 days at 20 °C (Figure 5f,g). Hyphal swellings doliiform, limoniform or elongate, mostly intercalary, sometimes terminal (Figure 5h). *Oogonia*: produced in single culture, globose to oval, smooth, sometimes with a small papilla, 15.3–24.6 µm in diameter (av. 21.2 µm), intercalary or terminal (Figure 5i–p). Oogonial stalks straight. Antheridia often 1–2 per oogonium, occasionally three per oogonium, globose, sac-shaped, clavate or allantoid, sometimes crook-necked, making apical or broad contact with oogonia, hypogynous, monoclinous, monoclinous sessile or diclinous (Figure 5i–o). Antheridial stalks unbranched. *Oospores*: plerotic or aplerotic, globose to subglobose, 13.7–21.7 µm in diameter (av. 17.7 µm), generally single, occasionally two in an oogonium, colorless with a wall 1.4–3.4 µm thick (av. 1.9 µm) (Figure 5i–p).

Other specimens examined: Dunnstown, Victoria, Australia, on root tissue of diseased *Tanacetum cinerariifolium*, June 2018, *A. Ahmed,* VPRI 43970; Dunnstown, Victoria, Australia, on root tissue of diseased *Tanacetum cinerariifolium*, June 2018, *A. Ahmed*, CBS 149751; Dunnstown, Victoria, Australia, on root tissue of diseased *Tanacetum cinerariifolium*, June 2018, *A. Ahmed,* VPRI 43972; Dunnstown, Victoria, Australia, on root tissue of diseased *Tanacetum cinerariifolium*, June 2018, *A. Ahmed*, VPRI 43973; Wynyard, Tasmania, Australia, on root tissue of diseased *Tanacetum cinerariifolium*, June 2020, *K. Groom*, UM2092; Wynyard, Tasmania, Australia, on root tissue of diseased *Tanacetum cinerariifolium*, June 2020, *K. Groom*, UM2094.

Known geographic distribution: Australia, Canada, Japan and the Netherlands.

Notes: *Globisporangium commune* belongs to *Globisporangium* genus *C* Uzuhashi, Kakishima and Tojo [27], and belongs to clade F *sensu* Lévesque and De Cock [15], related to *Globisporangium abappressorium* (Paulitz and M. Mazzola) Uzuhashi, Tojo and Kakish. This species is differentiated from *G. abappressorium* by the existence of single papilla on oogonia, and from other species of clade F by the unique sequences of nuclear ribosomal RNA and mitochondrial genes.

### 2.4. Pathogenicity Test

#### 2.4.1. In Vitro Pre-Germination Bioassays

**Figure 6:**
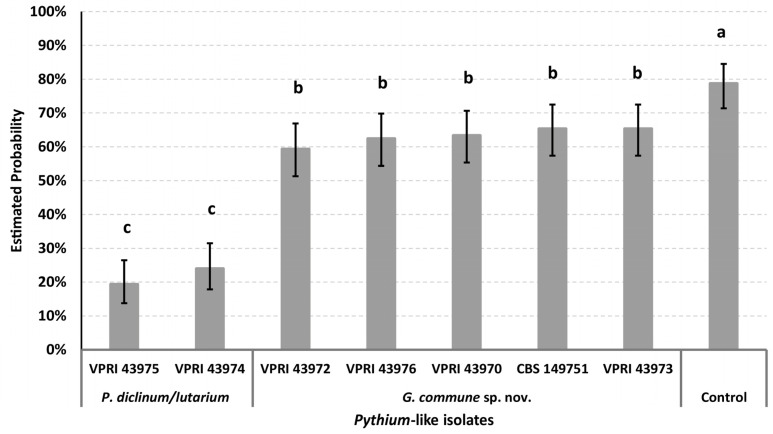
Predicted probability of pyrethrum seed germination inoculated with *Pythium diclinum/lutarium* and *Globisporangium commune* sp. nov. isolates, as calculated by the binary logistic regression model. Error bars show 95% confidence limit. Groups that do not share the same letter are significantly different by combination of odds ratios with 95% Wald confidence limits.

**Figure 7:**
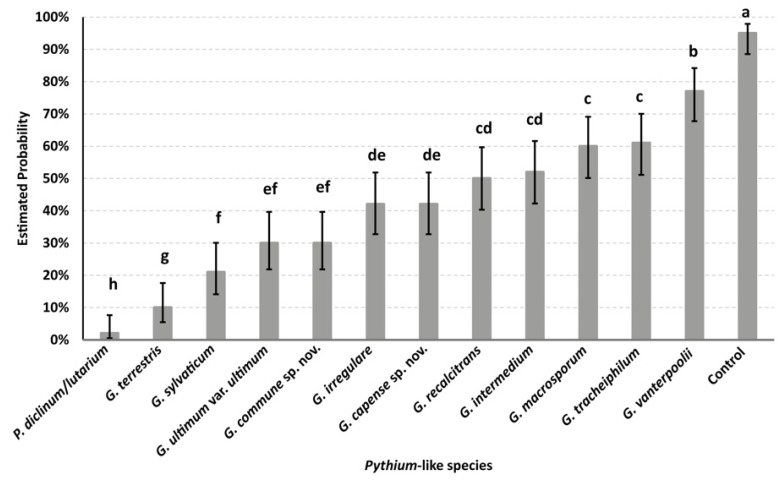
Predicted probability of pyrethrum seed germination inoculated with *Pythium* and *Globisporangium* species, as calculated by the binary logistic regression model. Error bars indicate 95% confidence limit. Groups that do not share the same letter are significantly different by combination of odds ratios with 95% Wald confidence limits.

Figure 6 and Figure 7 show the predicted probability of pyrethrum seed germination inoculated by *Pythium* and *Globisporangium* isolates, as calculated by the binary logistic regression models. The aggressiveness of *Pythium* and *Globisporangium* species varied among different species but was consistent within species.

In the first experiment, two isolates of *P. diclinum*/*lutarium* were significantly more aggressive than five *G. commune* sp. nov. isolates in terms of the effect on seed germination. Significant difference of aggressiveness was not observed within the same *Pythium* or *Globisporangium* isolates (Figure 6). All seeds in the control group fully germinated and grew into healthy seedlings, while seeds inoculated by *P. diclinum*/*lutarium* and *G. commune* sp. nov. were colonized by hyphae, and were necrotic before the emergence of their cotyledons.

In the second experiment, the analysis of seed germination showed that seeds inoculated with *P. diclinum*/*lutarium* had the lowest estimated probability of germination (<10%), followed by *G. terrestris*, *G. sylvaticum*, *G. ultimum* var. *ultimum*, *G. commune* sp. nov., *G. irregulare*, *G. capense* sp. nov., *G. recalcitrans* and *G. intermedium. G. macrosporum*, *P. tracheiphilum* and *P. vanterpoolii* were moderately aggressive on pyrethrum seeds, of which the estimated probability of germination was more than 60% (Figure 7).

For both germination experiments, the null hypothesis that the regression coefficients in the model are equal to zero was rejected (for the first experiment, *χ*_2_ = 163.72, df = 7, *p* < 0.0001; for the second experiment, *χ*_2_ = 205.67, df = 12, *p* < 0.0001), indicating that the effect of *Pythium*-like isolates on the germination of pyrethrum seed was significant. For the first and second experiments, the concordance statistics were 0.702 and 0.787, respectively (c value > 0.5), indicating both binary logistic regression model had high predictive power [28].

#### 2.4.2. Glasshouse Post-Germination Bioassays

All seedlings in control groups stayed healthy and were free from infection of any *Pythium* and *Globisporangium* species during experiments (Table 3).

In the first experiment, damping-off and seedling death occurred in every group within two weeks after being inoculated by *Pythium* and *Globisporangium* species, with average death rate from 4.2% to 35%. Of these, the *G. irregulare* group had the highest average death rate (DR = 35%), followed by *P. tracheiphilum* (30%), *G. commune* sp. nov. (27.5%). *G. sylvaticum, G. terrestris* and *G. recalcitrans*, which caused a moderate amount of seedling death (DR from 16.7% to 18.4%), while *G. capense* sp. nov., *G. intermedium, G. macrosporum* and *P. diclinum/lutarium* were the least aggressive in terms of causing seedling death (DR from 4.2% to 10%). All tested *Globisporangium* and *Pythium* species were recovered from inoculated plants. The average disease incidence ranged from 30% to 90.9%. *Globisporangium irregulare* had the highest average disease incidence (90.9%), followed by *G. terrestris* (85.9%)*, G. commune* sp. nov. (80.9%), *G. sylvaticum* (80%) and *P. diclinum*/*lutarium* (80%). Overall, the results of death rate and disease incidence largely reflected each other, except for *G. tracheiphilum* and *P. diclinum*/*lutarium. Pythium tracheiphilum* caused high average death rate (30%), but low average disease incidence (30%). Conversely, seedlings inoculated with *P. diclinum/lutarium* had the lowest average death rate (4.2%), but 80% were infected.

Compared to the control group, in Experiment 1 both above-ground and below-ground dry weight was significantly reduced six weeks after being inoculated by *G. irregulare*, *G. macrosporum*, *G. sylvaticum*, *G. terrestris*, *G. commune* sp. nov. and *G. recalcitrans* (Table 3). Of these species, *G. irregulare*, *G. macrosporum* and *G. sylvaticum* were the most aggressive in terms of causing dry weight reduction. None of the tested *Pythium* species were able to significantly reduce the dry weight of pyrethrum plants.

In the second experiment, 100% of pyrethrum seedlings (20 seedlings in total) were infected by *G. ultimum* var. *ultimum.* The death rate was 100% in Trial 1 and 60% in Trial 2. The seedlings first showed wilting and crown rot, then finally collapsed within two weeks after inoculation, which revealed that *G. ultimum* var. *ultimum* was highly aggressive on seedlings of pyrethrum BR2 variety.

## 3. Discussion

*Pythium* and *Globisporangium* species were isolated from poorly regrown pyrethrum plants in fields. Ten known *Globisporangium* species, two new *Globisporangium* species and three *Pythium* species were isolated from the root, crown and surrounding soils of symptomatic pyrethrum plants collected from 16 sites in Tasmania and Victoria. This is the first report of *Globisporangium* and *Pythium* species isolated from pyrethrum globally. In addition, this is the first report of *G. attrantheridium*, *G. macrosporum* and *G. terrestris* occurring in Australia. Globally, *G. macrosporum* has been reported as a pathogen of soybean and common bean [29,30] and *P. terrestris* has also been reported to cause losses in soybean [31]. *G. attrantheridium* has been reported in association with carrot cavity-spot lesion, and corn and soybean seed rot and seedling damping-off [32,33]. *Globisporangium intermedium*, *G. irregulare, G. recalcitrans G. rostratifingens*, *G. sylvaticum*, *G. ultimum* var. *ultimum, P. tracheiphilum* and *P. vanterpoolii* have previously been reported in Australia in association with processing tomato, parsley and parsnip crops in Victoria [9,10]. Two new pathogenic species, *Globisporangium capense* sp. nov. and *Globisporangium commune* sp. nov., were identified using phylogenetic studies. Their pathogenicity to cause seed rot and damping-off of pyrethrum was confirmed. Apart from Australia, *G. capense* sp. nov. was recorded as *Pythium* sp. from Canada from an unknown source [17]; and *G. commune* sp. nov. as *Pythium* sp. from Canada, Japan and the Netherlands from unknown sources [17].

In vitro pre-germination and glasshouse post-germination bioassays effectively assessed the pathogenicity of *Globisporangium* and *Pythium* species on pyrethrum seeds and seedlings. *Globisporangium* and *Pythium* species caused varying degrees of pyrethrum seed rot. As well as *G. ultimum* var. *ultimum* (average DR = 80%), six *Globisporangium* species also significantly reduced pyrethrum seed germination, caused post-germination seedling damping-off (average DR = 8.5–35%) and significantly reduced above-ground and below-ground dry weight of pyrethrum seedlings. Of these pathogenic *Globisporangium* species, *G. irregulare* and *G. ultimum* var. *ultimum* were the most aggressive on pyrethrum overall. However, *G. ultimum* var. *ultimum* was only isolated from soil in the fields. One explanation for the absence of species in living plants in the monitored fields is that this highly aggressive necrotrophic pathogen kills infected pyrethrum plants rapidly under natural field conditions and survives as a saprophyte in soil. This could also explain why it was only isolated from soil in the fields. Similarly, in a study conducted by Reeleder and Brammall [34], *G. ultimum*, which was only isolated from soil and not from ginseng plants, was shown to cause damping-off on inoculated ginseng seedlings. Both *G. irregulare* and *G. ultimum* var. *ultimum* can cause damping-off and root rot on a wide range of hosts, with both having been recorded as pathogens on more than 40 species of plant hosts in Australia [31,35,36,37]. *Globisporangium irregulare* and *G. ultimum* var. *ultimum* have also been reported to be pathogenic to some of the rotation crops and weeds that occur in pyrethrum cultivation sites, such as canola, carrot, cereal, some legume species, potato and subterranean clover [31,35,36,37,38,39]. These hosts in fields can also harbor other *Globisporangium* species identified from pyrethrum, such as *G. intermedium, G. recalcitrans* and *G. sylvaticum.* Thus, reducing inoculum of these *Globisporangium* species in the soil through rotation may be difficult due to the broad host range of some of these species. Although the two tested pyrethrum varieties were susceptible to *Pythium* and *Globisporangium* species, more pyrethrum varieties need to be evaluated for their resistance to these species as a possible control method.

The results of in vitro seed tests largely reflected the pathogenicity of *Pythium* and *Globisporangium* species on seedlings in pot trials. However, there were some inconsistencies in terms of the ranking of relative aggressiveness across the tests and plant growth stages. For instance, *P. diclinum/lutarium* was most aggressive in causing seed rot, but compared to the most aggressive species (*G. ultimum* var. *ultimum*, *G. macrosporum*, *G. irregulare* and *G. sylvaticum*), this species only moderately reduced biomass of pyrethrum seedlings and had low average DR (4.2%). Likewise, Matthiesen et al. [40] found that *P. lutarium* was more aggressive on soybean and corn in causing seed rot than causing root rot. Similar results were also observed by Rojas et al. [41] for *P. paroecandrum* and *P. spinosum*. Glasshouse post-germination bioassays verified that pathogenic *Pythium* and *Globisporangium* were able to cause biomass reduction and seedling damping-off in plants grown in potting mix. Tested species varied in their pathogenicity on pyrethrum seedlings, from non-pathogenic to pathogenic. Hence, instead of in vitro seed tests, glasshouse post-germination bioassays would be a more appropriate method for pyrethrum breeders to evaluate the susceptibility to *Globisporangium* and *Pythium.*

Previous studies suggested that *Pythium*-like species usually caused more severe damage at early stages of plant growth [14]. However, in pyrethrum fields, the incidence of *Pythium* and *Globisporangium* species from seedlings was low, whereas most of *Pythium* and *Globisporangium* species were isolated from pyrethrum plants showing stunting and brown discoloration of crown tissue in winter. One explanation for this is that disease incidence was greatly dependent on the inoculum level of *Pythium* and *Globisporangium* species [42,43], and the inoculum of these species in soils could be under a threshold level required to infect directly sown pyrethrum seeds and seedlings. Apart from this, the disease severity of *Pythium* and *Globisporangium* species is most likely related to external factors, such as temperature, soil properties, abiotic and biotic stresses on host plants and agronomic practices [14,44]. In Australia, pyrethrum is mainly cultivated on red ferrosols in northern Tasmania around Devonport and in Victoria around Ballarat, with average annual rainfall of 946 mm and 847 mm, respectively, and rainfall in winter is highest among the seasons, accounting for 34.5% and 30.9% of annual rainfall in each region, respectively [45]. Red ferrosols are known as well-drained soils with high organic matter levels, but continuous cultivation could result in significant structural degradation and fertility decline [46,47,48]. As a result, soil in pyrethrum cultivation regions could be cool and damp in winter, which favors the activity of many *Pythium*-like species, such as *G. irregulare, G. sylvaticum* and *G. ultimum* var. *ultimum* [33,49,50]. Since winter is the most possible season for disease outbreak of *Pythium*-like species, it is important to monitor pyrethrum plants for root rot in winter and monitor for heavy rains, especially in waterlogging-prone sites. Development of species-specific and sensitive Real-Time PCR assays may help to monitor inoculum levels of specific *Pythium* and *Globisporangium* species in pyrethrum fields.

Pyrethrum plants are perennial and exposed to a variety of abiotic and biotic stresses during their long establishment period. Pyrethrum is sensitive to waterlogging, and even a short period of waterlogging can cause severe growth reduction and aggravate infection by fungal pathogens [51,52]. Due to the complexity of soil biota, disease associated with soil-borne pathogens usually occurs within a disease complex. There are many reports of disease complexes involving *Pythium*-like species and *Fusarium* spp. [44,53,54]. *Fusarium oxysporum* isolated from diseased pyrethrum plants in yield-decline-affected fields can significantly reduce pyrethrum plant growth. However, in glasshouse pathogenicity trials, pyrethrum plants inoculated with only *F. oxysporum* did not show necrosis of the crown and roots although they were infected and had reduced biomass [4]. This could have been due to the need for several pathogens to infect together to produce a synergistic effect on disease development, such as may occur with *Fusarium* spp. and *Pythium*-like species. So far, little is known about the possible synergistic effect of soil-borne pathogens on disease levels of pyrethrum plants. Thus, further studies need to be conducted to confirm whether there is a synergistic relationship between *Pythium*-like species and *Fusarium* spp. associated with pyrethrum crops. These pathogens are prevalent in pyrethrum fields and could facilitate other fungi and oomycetes entering pyrethrum roots.

## 4. Materials and Methods

### 4.1. Sample Collection, Isolation and Preliminary Identification

From 2018 to 2021, *Pythium*-like isolates were obtained from pyrethrum plants collected from 16 sites in north Tasmania and the Ballarat region of Victoria, Australia. The sites ranged from fields with healthy plants to fields with severe poor regrowth (Supplementary Figure S2). The pyrethrum fields had variable cropping histories, ranging from no prior cultivation of pyrethrum to six years of pyrethrum cropping. In most sites, pyrethrum had been rotated with other crops, such as poppies, wheat, onions, tulips, canola, peas, potatoes and barley (Supplementary Table S1).

The first sampling was conducted in two sites near Dunnstown, Ballarat region in June 2018. The second sampling was conducted in March 2020 at two sites in north Tasmania. The third sampling was conducted in June 2020 at six sites in north Tasmania. The fourth and fifth samplings were carried out at the same six sites near Dunnstown, Ballarat region in December 2020 (at seedling stage) and November 2021 (right before the first harvest).

A total of 89 pyrethrum plants were sampled, including 54 asymptomatic plants and 35 plants showing stunting, wilting, poor growth, foliar chlorosis and black spots on leaves. Plants were arbitrarily selected and uprooted with intact crown, partial root systems and soil. Plant and soil samples were immediately transferred into plastic zip lock bags and stored at 4 °C for a maximum of two days before further processing. Soil attached to roots was removed and collected for soil baiting experiments, while the crown and root system of each plant was washed in running tap water to remove excess soil.

Root and crown tissues from each plant were surface-sterilized by immersion in 80% ethanol for 30 s, 1% ai NaOCl for 30 s to 2 min (depending on the size of tissues), rinsed twice in sterile deionized water (SDW) for 1 min and blotted with a sterile paper towel. Surface-sterilized tissues were cultured onto water agar (WA), potato dextrose agar (PDA) amended with Streptomycin (0.1 mg/mL) and V8-PAR oomycetes selective medium [22]. Plates were incubated at 24 °C for 2–3 days until hyphae were observed growing from tissues. Hyphae were subcultured onto PDA and incubated at 24 °C for a maximum of 7 days for preliminary morphological identification.

Two baiting techniques were used to isolate *Pythium*-like isolates from soil samples. For apple baiting, soil saturated with SDW was placed in three 2 cm deep holes, cut with a scalpel, inside the apple variety Granny Smith. Apple baits were sealed by parafilm and incubated at room temperature until symptom development. For the second technique, young leaves of *Eucalyptus cinerea* and white rose petals (*Rosa* sp.) were used as baits. Two teaspoons of soil were placed in open plastic containers and SDW was added until the depth of water was 5 cm. After mixing thoroughly, debris floating on the water surface was removed by sterile spoons and baits were floated on water surface. These containers were incubated at room temperature until the development of necrotic lesions on the leaves or petals. After the appearance of symptoms, margins of affected plant tissues were cut, surface-sterilized with 70% ethanol and placed on V8-PAR selective medium [22]. Plates were incubated at 24 °C for 2–3 days until hyphae were observed growing from the tissue. Hyphae were subcultured onto PDA and incubated at 24 °C for up to 7 days for preliminary morphological identification.

*Pythium*-like isolates were tentatively identified based on colony morphology. Pure cultures of tentative *Pythium*-like isolates were established by the hyphal tipping technique and cultured onto V8-PAR medium and accessioned with University of Melbourne (UM) numbers. Pure isolates were divided into different morphotypes according to observation of macroscopic (colony color and growth rate) and microscopic (hyphae and spores) features described by Van der Plaats-Niterink [14]. Cultures were submerged in twice-autoclaved tap water (~20 mL) containing six hemp (*Cannabis sativa*) seeds in McCartney bottles and stored in darkness at room temperature prior to further molecular identification. Representative cultures were deposited at the Victorian Plant Pathogen Herbarium (VPRI), Victoria, Australia, or at the Westerdijk Fungal Biodiversity Institute (CBS), Utrecht, the Netherlands.

### 4.2. Morphological Characterization of New Species

Cultures were examined morphologically to describe new species. Axenic isolates were cultivated on cornmeal agar (CMA), potato carrot agar (PCA), PDA and V8 at 24 °C for 14 days to observe sexual structures. The observation of sporangia and zoospore release was adapted from the method of De Cock and Lévesque [55]. Isolates were first subcultured on PDA. After 3 days, pieces of sterilized rye (*Secale cereale*) leaves were placed on colonies. After 1 day, leaves were colonized by mycelia and were transferred to 90 mm diameter Petri dishes containing sterile soil extract and were maintained at 15–20 °C for 14 days under natural sunlight to induce formation of sporangia and zoospores. The soil extract was prepared according to De Cock and Lévesque [55]. All cultures were checked after 3 days, and everyday thereafter. Agar plugs (5 mm^2^) of CMA cultures and mycelial mats formed in soil extract were transferred onto glass slides, mounted in water or lactophenol cotton blue for microscopical examination referring to morphological keys described by Van der Plaats-Niterink [14]. For each species, fifty oogonia and forty sporangia were measured with a Leica DM6000 B light microscope (Leica, Wetzlar, Germany), Leica DM450 C digital microscope camera (Leica, Wetzlar, Germany) and Leica LAS V4.12 software (Leica, Wetzlar, Germany) using X400 magnification.

The cardinal temperature and daily growth rates were examined on PCA with three replicate plates per species. For each species, an agar plug (5 mm^2^) of a 7-day-old culture was placed in the center of a 150 mm diameter Petri dish. The plates were placed at 24 °C for 24 h in the dark. Two perpendicular lines were drawn on the bottom of the plates and intersected at the place of agar plugs. Colony margins were then marked in all four directions on these lines. Plates were then incubated for a further 24 h at temperatures of 5 °C to 40 °C with intervals of 5 °C in the dark. Colony margins were marked again and the growth between two points on the line was measured. Daily radial growth was calculated by averaging all measurements of three replicate plates. Cultures that were able to grow under 5 °C were brought to lower temperatures of 4 °C, 3 °C, 2 °C, 1 °C and 0.5 °C to determine the minimum temperature required for growth. When no growth was observed before Petri dishes were fully covered by colonies, the culture was returned to 24 °C to check whether growth resumed.

### 4.3. Molecular Identification and Phylogenetics

A total of 52 representative *Pythium*-like isolates were selected for molecular identification and phylogenetic analysis with 50 reference isolates.

Mycelia of 7-day-old pure cultures on PDA were scraped from the surface of the agar, flash-frozen in liquid nitrogen and ground into a fine powder using a mortar and pestle. Genomic DNA was extracted using the DNeasy Plant Mini Kit (Qiagen, Melbourne, Australia) according to manufacturer’s protocol. The DNA concentrations were estimated using a NanoDrop™ OneC Microvolume UV-Vis Spectrophotometer (Thermo Scientific, Wilmington, USA). DNA integrity was assessed through gel electrophoresis using 1% *w*/*v* agarose gel stained with SYBR™ Safe DNA Stain (Invitrogen, Carlsbad, USA).

The ITS region of the nrDNA was amplified using primers ITS1 (5′-TCCGTAGGTGAACCTGCGG-3′) and ITS4 (5′-TCCTCCGCTTATTGATATGC-3′) [56]. Partial *Cox1* region was amplified using OomCoxI-Levup (5′-TCAWCWMGATGGCTTTTTTCAAC-3′) and OomCoxI-Levlo (5′-CYTCHGGRTGWCCRAAAAACCAAA-3′) [17] primer pair. The PCR mixture and cycle were adapted from the protocol described by Le, Smith and Aitken [8]. The final concentrations for a 20 µL reaction were 0.71X MangoTaq colorless reaction buffer (Bioline, Sydney, Australia), 0.525 mM MgCl_2_ (Bioline, Sydney, Australia), 0.375 µM of each primer, 1.25 ng/μL genomic DNA for amplification of ITS, 0.625 ng/μL genomic DNA for amplification of *Cox1*, 0.045 U MangoTaq polymerase (Bioline, Sydney, Australia) and sterile Milli-Q water to obtain a 20 µL final volume. The amplification reactions were performed in an Eppendorf thermal cycler (Eppendorf, Sydney, Australia) using the protocol described by Le et al. [57]. All amplified products were subjected to electrophoresis at 80 V in 1% *w*/*v* agarose gel stained with SYBR™ Safe DNA Gel Stain (Invitrogen, Carlsbad, CA, USA) for 40 min and visualized under UV light. Amplicons were purified by the QIAquick PCR purification kit (Qiagen, Melbourne, Australia) and submitted to the Australian Genome Research Facility (AGRF, Melbourne, Australia) for forward and reverse Sanger sequencing.

The quality of each forward- and reverse-direction sequence was checked using Chromas 2.6.6 software (Technelysium, Brisbane, Australia), aligned by ClustalW and manually trimmed using MEGAX software [58]. Consensus sequences were subjected to Basic Local Alignment Search Tool (BLAST) in the National Center for Biotechnology Information (NCBI) GenBank database (National Center for Biotechnology Information 2020) for putative identification of species.

Before construction of the phylogenetic tree, identical duplicate sequences in the dataset were identified. To reduce the computation time and declutter the appearance of the tree, only one of each set of identical sequences was selected as a representative, and the rest of the duplicate sequences were deleted. For each locus, sequences were aligned with the closely associated reference sequences derived from the GenBank database (Table 4) by using the MAFFT plugin in Geneious Prime 2022.2.2 (https://www.geneious.com accessed on 29 November 2022). Consensus ITS and *Cox1* sequences were deposited in the GenBank database.

In order to depict phylogenetic relationships between sequenced isolates and known reference strains of *Globisporangium* and *Pythium* species, phylogenetic trees were constructed for each gene separately, and also for concatenated alignments of combined ITS and *Cox1* using the Maximum Likelihood (ML) statistical method in MEGAX software. The best-fit model HKY + G was selected by MEGAX software to build the phylogenetic tree of the ITS locus, while GTR + G + I was selected for the rest of the datasets. The tree inference option was Nearest-Neighbor-Interchange (NNI) ML heuristic method. Gaps were treated with partial deletion with site coverage cut-off of 95%. The confidence of the branches in the ML tree was estimated by bootstrap analysis with 1000 replicates.

Bayesian Inference (BI) analyses were conducted by MrBayes version 3.2.7a [59] for each gene separately, and for concatenated alignments of combined ITS and *Cox1*. MrModeltest2 [60] was used to determine the best-fit model for each locus for BI analysis. Model test analysis resulted in a HKY + G + I model for ITS locus region, while for *Cox1* it produced a GTR + G + I model. Four chains were used in the Markov Chain Monte Carlo analysis with a temperature value of 0.2. The number of generations and sample frequencies were set, respectively, at 1,000,000,000 and 100. Analyses stopped once the value of the convergence diagnostic was below 0.01. The first 25% of generated trees were excluded as the ‘burn-in’ phase and posterior probabilities (PP) were calculated based on the remaining trees.

### 4.4. Pathogenicity Test

#### 4.4.1. In Vitro Pre-Germination Bioassays

Two experiments were conducted to assess the pathogenicity of *Pythium* and *Globisporangium* species on pyrethrum seeds. The term pathogenicity here is defined as the ability of isolates to infect and reduce biomass of pyrethrum plants. Pyrethrum seeds were provided by Botanical Resources Australia. In the first experiment, the pathogenicity of different isolates was compared within two *Pythium*-like species (*G. commune* sp. nov. and *P. diclinum/lutarium*). This experiment was carried out twice. The second experiment was conducted similarly to the first experiment with more *Pythium* and *Globisporangium* isolates. This experiment included representative isolates of 12 *Pythium* species. The experiment was conducted twice. The details of each experiment are listed in Table 5.

For both experiments, a 0.5 × 0.5 cm mycelial agar plug was cut from the PDA culture of each 7-day-old *Pythium*-like isolate and placed on the center of WA plates. The WA plates were sealed and incubated at 24 °C and 12 h photoperiod for 7 days. Disease-free pyrethrum seeds were firstly surface-sterilized by being immersed in 80% ethanol for 30 s, and 1% ai NaOCl for 1 min, rinsed twice in SDW for 1 min and blotted with a sterile paper towel. Ten surface-sterilized pyrethrum seeds (variety RS5 in the first experiment and BR2 in the second experiment) were placed evenly around the mycelial plug on the mycelia of the surface of the WA and incubated at 24 °C for a 12 h photoperiod. The control treatment had a blank PDA plug without any isolates per plate. Each treatment included five or ten replicated plates.

Germinated seeds were counted at 28 days after culturing. Germination of seed is defined as the stage when the radicle emerges partially or completely from the seed coat. Because germination of pyrethrum seed was a binary response (germination and non-germination), probability of germination of each treatment was assessed using binary logistic regression using the model:

$$Y_{i}\;\sim \;Binominal\left(1,\;p_{i}\right),\;\log\left(\frac{p_{i}}{1-p_{i}}\right)=\beta_{0}+\beta_{1}X_{i}$$

where *Y* is the germination status of each individual seed (*i*), which has a binomial distribution with probability *p*. *β*_0_ is an intercept parameter and *β*_1_ is the coefficient for the effect of *Pythium*-like isolate (*X*). Significance of the parameters of the model was evaluated using the logistic procedure in SAS version 9.4 (SAS Institute, Cary, NC, USA). Individual models were fitted for each experiment. The effect of the trial and the effect of the plate were not included in the model because these two factors were found to be not significant (*p* > 0.05). Interaction between any factors was not found (*p* > 0.05).

#### 4.4.2. Glasshouse Post-Germination Bioassays

Two experiments were conducted to evaluate the pathogenicity of 11 species on pyrethrum seedlings (variety BR2) in pots. Each experiment was conducted twice in a completely randomized design.

- Experiment 1

In the first experiment, all pyrethrum seedlings were maintained in a glasshouse at 20–24 °C under natural light with ten *Pythium* and *Globisporangium* species inoculations as treatments. There were ten treatments (one representative isolate per species) and one control. Each treatment included twelve replicates in Trial 1 and ten replicates in Trial 2. The details of treatment are listed in Table 6.

Millet seed inocula of *Pythium* and *Globisporangium* isolates were made as described by Callaghan, Burgess, Ades, Tesoriero and Taylor [10]. An 8-week-old pyrethrum seedling was transplanted into a surface-sterilized pot (600 mL) filled with steam-pasteurized potting mix which contained 5% (*w*/*v*) inoculated millet seed. Pots were arranged in a completely randomized design and observed daily. The control treatments were the equivalent amount of sterile millet. Dead seedlings were recorded, removed and cultured onto WA and then PDA to confirm *Pythium* and *Globisporangium* species infection.

Six weeks after inoculation, surviving seedlings were destructively sampled to assess for *Pythium* and *Globisporangium* species infection. Seedlings were uprooted carefully to keep intact crown and root systems. Tissues from the petiole base, crown and upper root of each plant were randomly selected and cut into 0.5 cm pieces, and immersed in 1% ai NaOCl for 20 s, rinsed two times in SDW for 1 min and blotted with a sterile paper towel. These pieces of tissue were placed on WA and incubated at 24 °C in a 12 h photoperiod. Hyphal tips growing on WA were subcultured onto PDA amended with Streptomycin (0.1 mg/mL) and incubated for 5–7 days at 24 °C to confirm presence of the pathogen. Identification of the *Pythium*-like strains that emerged was based on colony morphology on PDA and micromorphological features [14].

Plants were dried in an oven at 71 °C for three days. Above-ground and below-ground biomass of the harvested plants were measured to compare the effect of different *Pythium* and *Globisporangium* species on pyrethrum plants. Disease incidence (DI) for each group was calculated by

$$Disease\;incidence\;\left(\mathrm{DI}\right)=\frac{Number\;of\;dead\;plants+Number\;of\;infected\;living\;plants}{Total\;number\;of\;assessed\;plants}.$$

Death rate (DR) for each group was calculated by

$$Death\;rate\;\left(\mathrm{DR}\right)=\frac{Number\;of\;dead\;plants}{Total\;number\;of\;assessed\;plants}.$$

Data were analyzed by using Minitab 19.1.0 software (Minitab, Sydney, Australia) by general linear model. All trials were combined in one analysis, using the model:

$$Response=overall\;mean+treatment\;effect+trial\;effect+error,\;\;Y_{ij}=\mu +\tau_{i}+\gamma_{j}+\varepsilon_{ij}\;$$

where *µ* is the overall mean, *τ_i_* is the effect of treatment *i*, *γ_j_* is the effect of trial *j* and *ε_ij_* is an independent random error. The *ε* is assumed to be normally distributed with the same standard deviation and independent. If the *p*-value was less than 0.05, transformed means were used to compare groups by using Tukey’s Pairwise Comparison test (α = 0.05). Due to the unequal variance of residuals, log transformation was used in dry weight data analysis of glasshouse post-germination bioassay.

- Experiment 2

Experiment 2 was conducted in a plant growth chamber at 20–24 °C under a 12/12 light/dark cycle, with inoculum of *G. ultimum* var. *ultimum* isolate VPRI 43979 as treatment. Each treatment included ten replicates (ten pots). For each pot, an 8-week-old pyrethrum seedling was inoculated as described in Experiment 1 of glasshouse post-germination bioassays. Pots were arranged in a completely randomized design and observed daily. Dead seedlings were recorded, removed and cultured onto WA and then PDA to confirm *G. ultimum* var. *ultimum* infection. Because most pyrethrum seedlings died within 14 days after inoculation, only disease incidence and death rate were calculated and analyzed as described in Experiment 1 of glasshouse post-germination bioassays.

## 5. Conclusions

These results indicate that *Pythium*-like species are commonly isolated from diseased pyrethrum plants and may contribute to the pyrethrum yield decline situation in Australia. These findings improve understanding of the disease complex associated with pyrethrum yield decline and provide useful information to develop sustainable management strategies.

## Figures and Tables

**Table 1:** Details of *Pythium* and *Globisporangium* species isolated from pyrethrum plants or soil.

*Pythium* and *Globisporangium* Species ^a^	Dates Sampled	Total Number of Isolates ^b^	Number of Sites *Pythium* and *Globisporangium* spp. Isolated	Substrate ^c^	Cultures Deposited at VPRI or CBS
*G. attrantheridium*	November 2021	1	1	R	CBS 149757
*G. erinaceum*	November 2021	1	1	R	CBS 149756
*G. intermedium*	June 2020	4	2	C, R	VPRI 43980
*G. irregulare*	June 2018; June 2020	4	4	C, R, S	VPRI 43981; VPRI 43977
*G. macrosporum*	June 2020	1	1	S	VPRI 43989
*G. recalcitrans*	June 2020	3	2	R	VPRI 43986
*G. rostratifingens*	December 2020	1	1	C	CBS 149755
*G. capense* sp. nov.	June 2020	1	1	R	CBS 149752
*G. commune* sp. nov.	June 2018; June 2020	8	4	R, S	CBS 149753; VPRI 43970; CBS 149753; VPRI 43972; VPRI 43973; VPRI 43976
*G. sylvaticum*	June 2020	10	4	R, S	VPRI 43982
*G. terrestris*	June 2020	1	1	R	VPRI 43987
*G. ultimum* var. *ultimum*	March 2020; June 2020; November 2021	17	7	S	VPRI 43979
*P. diclinum/lutarium*	June 2018; June 2020	3	2	R, S	VPRI 43984; VPRI 43974; VPRI 43975;
*P. tracheiphilum*	June 2020	5	2	C, R	CBS 149754
*P. vanterpoolii*	June 2018; June 2020	2	2	C, S	VPRI 43988; VPRI 43978

^a^ Species determined by morphology and sequence-based similarity (ITS and *Cox1*); ^b^ isolated from different pieces of plant tissue; ^c^ C = crown; R = root; S = soil.

**Table 2:** *Pythium* and *Globisporangium* spp. isolated from the surveyed pyrethrum sites.

Time Sampled	Site Number	Location	Plants Sampled and Comments on Sites	Number of Isolates	*Pythium* and *Globisporangium* Species (Number of Isolates)
June 2018	91808; 92012	Dunnstown, VIC	For each site, three symptomatic plants were randomly sampled.	9	*G. irregulare* (1); *P. lutarium/diclinum* (2); *G. commune* sp. nov. (5); *P. vanterpoolii* (1)
March 2020	46604; 46026	Penguin, TAS	Plants were healthy. For each site, three asymptomatic plants and surrounding soil were randomly sampled.	7	*G. ultimum* var. *ultimum* (7)
June 2020	63711; 874020; 700091; 700090; 70087; 646009	Wynyard, TAS	Plants ranged from healthy to severe poor regrowth. For each site, three symptomatic plants were randomly sampled.	38	*G. intermedium* (4); *G. irregulare* (1); *P. lutarium/diclinum* (1); *G. macrosporum* (1); *G. recalcitrans* (3); *G. capense* sp. nov. (1); *G. commune* sp. nov. (3); *G. sylvaticum* (10); *G. terrestris* (1); *P. tracheiphilum* (5); *G. ultimum* var. *ultimum* (7); *P. vanterpoolii* (1)
December 2020	902015; 910013; 918012; 922013; 949006; 954001	Dean, VIC; Dunnstown, VIC; Mollongghip, VIC; Bullarook, VIC	Four-month-old seedlings on this site were healthy. For each site, five healthy plants and their surrounding soil were randomly sampled.	1	*G. rostratifingens* (1)
November 2021	902015; 910013; 918012; 922013; 949006; 954001	Dean, VIC; Dunnstown, VIC; Mollongghip, VIC; Bullarook, VIC	Plants were 15 months old and ready for harvest. All sites looked uniform and healthy overall, with a few plants showing wilting and stunting. For each site, three asymptomatic plants and their surrounding soil were randomly sampled. Eleven plants showing disease symptoms were sampled from five sites.	7	*G. attrantheridium* (1); *G. erinaceum* (1); *G. irregulare* (2); *G. ultimum* var. *ultimum* (3)

**Table 3:** Average disease incidence, death rate and dry weight of pyrethrum seedlings six weeks after inoculation with *Pythium* and *Globisporangium* species. In the same column, means of dry weight that do not share the same letter are significantly different by combination of General Linear Model and Tukey’s Pairwise Comparison test (α = 0.05).

Experiment	Treatment	*Globisporangium* and *Pythium* spp.	Tissues Infected ^b^	Average Disease Incidence (DI)	Average Death Rate (DR)	Dry Weight Component (g) ^a^	
						Above-Ground	Below-Ground
Experiment 1	Control			0%	0%	2.84 a	0.62 a
	VPRI 43980	*G. intermedium*	C, R	57.5%	8.4%	2.27 ab	0.37 ab
	UM2130	*P. tracheiphilum*	C, R	30%	30%	2.36 abc	0.52 ab
	VPRI 43984	*P. diclinum/lutarium*	C, R	80%	4.2%	1.66 abc	0.29 ab
	CBS 149752	*G. capense* sp. nov.	C, R	63.4%	10%	1.47 abc	0.25 ab
	VPRI 43986	*G. recalcitrans*	C, R	30.9%	16.7%	1.37 bc	0.24 b
	CBS 149753	*G. commune* sp. nov.	C, R	80.9%	27.5%	1.13 cd	0.21 bc
	VPRI 43987	*G. terrestris*	C, R	85.9%	18.4%	0.89 cd	0.13 bc
	VPRI 43982	*G. sylvaticum*	C, R	80%	18.4%	0.62 de	0.06 d
	VPRI 43981	*G. irregulare*	C, R	90.9%	35%	0.49 e	0.06 cd
	VPRI 43989	*G. macrosporum*	C, R	57.5%	8.4%	0.56 e	0.09 d
						*p*-value < 0.0001	*p*-value < 0.0001
Experiment 2	Control			0%	0%	N/A	N/A
	VPRI 43979	*G. ultimum* var. *ultimum*	C, R	100%	80%	N/A	N/A

^a^ Log-transformed means were used to compare groups. ^b^ C = crown; R = root.

**Table 4:** Information about the *Pythium* and *Globisporangium* spp. isolates used in this study.

*Pythium*-like Species	Isolate Accession No.	Location	Isolate Source	GenBank Accession No.	
				ITS	*Cox1*
G. abappressorium *	CBS 110198	USA	*Triticum aestivum*	HQ643408.2	HQ708455
*G. abappressorium*	Isolate 020162	USA	Soil (*Triticum* sp.)	DQ091294	-
*G. abappressorium*	Isolate 131	USA	Soil	MK886852	-
*G. abappressorium*	Isolate 216	USA	Soil	MK886853	-
*G. abappressorium*	Isolate 712	USA	Soil	MK886854	-
G. attrantheridium *	DAOM 230386	Unknown	Unknown	HQ643476.2	HQ708523
***G. attrantheridium***	**CBS 149757**	Australia	*Tanacetum cinerariifolium*	**OM780318**	-
***G. capense* sp. nov.**	**CBS 149752**	Australia	*T. cinerariifolium*	**OL342598**	**OL331986**
*G. capense* sp. nov.	BR879	Canada	Unknown	HQ643817	HQ708858
***G. commune* sp. nov.**	**UM2092**	Australia	*T. cinerariifolium*	**OL342600**	**OL331988**
***G. commune* sp. nov.**	**CBS 149753**	Australia	*T. cinerariifolium*	**OL342601**	**OL331989**
***G. commune* sp. nov.**	**UM2094**	Australia	*T. cinerariifolium*	**OL342602**	**OL331990**
***G. commune* sp. nov.**	**VPRI 43973**	Australia	*T. cinerariifolium*	**OL952620**	**OL860922**
***G. commune* sp. nov.**	**VPRI 43970**	Australia	*T. cinerariifolium*	**OL952617**	**OL860919**
***G. commune* sp. nov.**	**CBS 149751**	Australia	*T. cinerariifolium*	**OL952618**	**OL860920**
***G. commune* sp. nov.**	**VPRI 43972**	Australia	*T. cinerariifolium*	**OL952619**	**OL860921**
*G. commune* sp. nov.	ADC9982	The Netherlands	Unknown	HQ643827	HQ708868
*G. commune* sp. nov.	BR902	Canada	Unknown	HQ643811	HQ708852
*G. commune* sp. nov.	Lev3106	Japan	Unknown	HQ643798	HQ643798
*G. cryptoirregulare*	CBS 118731	USA	*Euphorbia pulcherima*	HQ643515.2	GU071825
G. cylindrosporum *	CBS 218.94	Germany	Soil	HQ643516	HQ708562
*G. debaryanum*	CBS 752.96	UK	*Tulipa* sp.	HQ643519	HQ708565
G. erinaceum *	CBS 505.80	New Zealand	Soil	HQ643534	HQ708578
***G. erinaceum***	**CBS 149756**	Australia	*T. cinerariifolium*	**OM780317**	-
G. heterothallicum *	CBS 450.67	Canada	*Sambucus* sp.	HQ643553	HQ708597
G. intermedium *	CBS 266.38	The Netherlands	*Agrostis stolonifera*	HQ643572	HQ708616
***G. intermedium***	**UM2128**	Australia	*T. cinerariifolium*	**OL342577**	**OL331964**
***G. intermedium***	**UM2129**	Australia	*T. cinerariifolium*	**OL342578**	**OL331965**
***G. intermedium***	**VPRI 43980**	Australia	*T. cinerariifolium*	**OL342595**	**OL331983**
***G. intermedium***	**UM2098**	Australia	*T. cinerariifolium*	**OL342606**	**OL331994**
***G. irregulare***	**VPRI 43977**	Australia	Soil (*T. cinerariifolium*)	**OL952624**	**OL860926**
G. irregulare *	CBS 250.28	The Netherlands	*Phaseolus vulgaris*	HQ643596	HQ708640
***G. irregulare***	**VPRI 43981**	Australia	*T. cinerariifolium*	**OL342596**	**OL331984**
G. kunmingense *	CBS 550.88	China	*Vicia faba*	HQ643672	HQ708716
*G. lucens*	CBS 113342	Unknown	*Triticum* sp.	HQ643681	HQ708725
G. macrosporum *	CBS 574.80	The Netherlands	Flower bulb	HQ643684	HQ708728
***G. macrosporum***	**VPRI 43989**	Australia	Soil (*T. cinerariifolium*)	**OL342583**	**OL331971**
G. paroecandrum *	CBS 157.64	Australia	Soil	HQ643731	HQ708772
***G. recalcitrans***	**UM2106**	Australia	*T. cinerariifolium*	**OL342574**	**OL331962**
*G. recalcitrans*	Py26	Mallorca (Spain)	*Beta vulgaris*(roots)	DQ357833	EF426549
***G. recalcitrans***	**VPRI 43986**	Australia	*T. cinerariifolium*	**OL342603**	**OL331991**
***G. recalcitrans***	**UM2096**	Australia	*T. cinerariifolium*	**OL342604**	**OL331992**
G. rostratifingens *	CBS 115464	USA	Soil	HQ643761.2	HQ708802
***G. rostratifingens***	**CBS 149755**	Australia	*T. cinerariifolium*	**OL342594**	**OM807117**
G. spiculum *	CBS 122645	France	Soil	HQ643790	HQ708831
G. spinosum *	CBS 276.67	The Netherlands	*Zinnia* sp.	HQ643793	HQ708834
G. splendens *	CBS 462.48	USA	Unknown	HQ643795	HQ708836
***G. sylvaticum***	**UM2111**	Australia	*T. cinerariifolium*	**OL342575**	**OL331963**
***G. sylvaticum***	**UM2140**	Australia	*T. cinerariifolium*	**OL342579**	**OL331966**
***G. sylvaticum***	**UM2171**	Australia	*T. cinerariifolium*	**OL342580**	**OL331968**
***G. sylvaticum***	**UM2186**	Australia	Soil (*T. cinerariifolium*)	**OL342581**	**OL331969**
***G. sylvaticum***	**UM2187**	Australia	Soil (*T. cinerariifolium*)	**OL342582**	**OL331970**
***G. sylvaticum***	**UM2190**	Australia	Soil (*T. cinerariifolium*)	**OL342585**	**OL331973**
***G. sylvaticum***	**UM2192**	Australia	Soil (*T. cinerariifolium*)	**OL342586**	**OL331975**
***G. sylvaticum***	**UM2193**	Australia	Soil (*T. cinerariifolium*)	**OL342587**	**OL331976**
***G. sylvaticum***	**UM2194**	Australia	Soil (*T. cinerariifolium*)	**OL342588**	**OL331977**
*G. sylvaticum*	STE-U6800	Unknown	Unknown	GQ410350	GU071816
G. sylvaticum *	CBS 453.67	USA	*Prunus persica*	HQ643845	HQ708886
***G. sylvaticum***	**VPRI 43982**	Australia	*T. cinerariifolium*	**OL342597**	**OL331985**
*G. terrestris*	ADC9906	The Netherlands	Unknown	HQ643858	HQ708899
G. terrestris *	CBS 112352	France	Soil	HQ643857	HQ708898
***G. terrestris***	**VPRI 43987**	Australia	*T. cinerariifolium*	**OL342605**	**OL331993**
*G. ultimum* var. sporangiiferum *	CBS 219.65	USA	Unknown	HQ643879	HQ708920
***G. ultimum* var. *ultimum***	**UM2189**	Australia	Soil (*T. cinerariifolium*)	**OL342584**	**OL331972**
***G. ultimum* var. *ultimum***	**UM2196**	Australia	Soil (*T. cinerariifolium*)	**OL342589**	**OL331978**
***G. ultimum* var. *ultimum***	**UM2197**	Australia	Soil (*T. cinerariifolium*)	**OL342590**	**OL331979**
***G. ultimum* var. *ultimum***	**UM2198**	Australia	Soil (*T. cinerariifolium*)	**OL342591**	**OL331980**
***G. ultimum* var. *ultimum***	**UM2200**	Australia	Soil (*T. cinerariifolium*)	**OL342592**	**OL331981**
***G. ultimum* var. *ultimum***	**UM2201**	Australia	Soil (*T. cinerariifolium*)	**OL342593**	**OL331982**
***G. ultimum* var. *ultimum***	**UM2338**	Australia	Soil (*T. cinerariifolium*)	**OL342607**	**OL331995**
***G. ultimum* var. *ultimum***	**UM2339**	Australia	Soil (*T. cinerariifolium*)	**OL342608**	**OL331996**
***G. ultimum* var. *ultimum***	**UM2340**	Australia	Soil (*T. cinerariifolium*)	**OL342609**	**OL331997**
***G. ultimum* var. *ultimum***	**UM2341**	Australia	Soil (*T. cinerariifolium*)	**OL342610**	**OL331998**
***G. ultimum* var. *ultimum***	**UM2342**	Australia	Soil (*T. cinerariifolium*)	**OL342611**	**OL331999**
***G. ultimum* var. *ultimum***	**VPRI 43979**	Australia	Soil (*T. cinerariifolium*)	**OL342612**	**OL332000**
***G. ultimum* var. *ultimum***	**UM2344**	Australia	Soil (*T. cinerariifolium*)	**OL342612**	**OL332001**
*G. ultimum* var. ultimum *	CBS 398.51	The Netherlands	*Lepidium sativum*	HQ643865	HQ708906
*G. ultimum* var. *ultimum*	BR144	Canada	Unknown	HQ643943	HQ708984
*G. ultimum* var. *ultimum*	BR793	South Africa	Unknown	HQ643913	HQ708954
*G. ultimum* var. *ultimum*	BR816	South Africa	Unknown	HQ643909	HQ708950
G. viniferum *	CBS 119168	France	Soil (*Vitis* sp.)	HQ643956	HQ708997
*Globisporangium* sp.	ADC9966	The Netherlands	Unknown	HQ643828	HQ708869
*Globisporangium* sp.	Lev1457	Canada	Unknown	HQ643804	HQ708845
P. aphanidermatum *	CBS 118.80	Unknown	Unknown	HQ643438	HQ708485
P. coloratum *	CBS 154.64	Australia	Soil	HQ643501	HQ708547
P. deliense *	CBS 314.33	Sumatra	*Nicotiana tabacum*	HQ643522	HQ708568
P. diclinum *	CBS 664.79	The Netherlands	*B. vulgaris*	HQ643524	HQ708570
***P. diclinum/lutarium***	**VPRI 43975**	Australia	*T. cinerariifolium*	**OL952622**	**OL860924**
***P. diclinum/lutarium***	**VPRI 43974**	Australia	*T. cinerariifolium*	**OL952621**	**OL860923**
***P. diclinum/lutarium***	**VPRI 43984**	Australia	*T. cinerariifolium*	**OL342599**	**OL331987**
P. dissotocum *	CBS 166.68	USA	Unknown	HQ643528	HQ708574
P. inflatum *	CBS 168.68	USA	*Saccharum officinarum*	HQ643566	HQ708610
P. lutarium *	CBS 222.88	UK	Soil	HQ643682	HQ708726
P. phragmitis *	CBS 117104	Germany	*Phragmites australis*	HQ643746	HQ708787
P. torulosum *	CBS 316.33	The Netherlands	Grass root	HQ643859	HQ708900
***P. tracheiphilum***	**CBS 149754**	Australia	*T. cinerariifolium*	**OL342576**	**OM807115**
***P. tracheiphilum***	**UM2130**	Australia	*T. cinerariifolium*	**OM780315**	**OM807116**
P. tracheiphilum *	CBS 323.65	Italy	*Lactuca sativa*	HQ643862	HQ708903
***P. vanterpoolii***	**VPRI 43978**	Australia	Soil (*T. cinerariifolium*)	-	**OL860927**
***P. vanterpoolii***	**VPRI 43988**	Australia	*T. cinerariifolium*	-	**OL331967**
P. vanterpoolii *	CBS 295.37	UK	*T. aestivum*	HQ643952	HQ708993
Phytophthora capsici *	CBS 128.23	USA	*Capsicum annuum*	HQ643180	HQ708249

Isolates in this study are in bold. Asterisks on isolate names denote type-derived material or strains used by Van der Plaats-Niterink [14] for species descriptions.

**Table 5:** In vitro pre-germination bioassays of pyrethrum seed inoculated with *Globisporangium* and *Pythium* spp.

Experiment	Trial	Pyrethrum Variety	Isolates	Replicates
Experiment 1	Trial 1/Trial 2	RS5	7 isolates *G. commune* sp. nov. (VPRI 43970, CBS 149753, VPRI 43972, VPRI 43973, VPRI 43976) *P. diclinum/lutarium* (VPRI 43974, VPRI 43975)	10 plates (Trial 1) 5 plates (Trial 2) 10 seeds/plate
Experiment 2	Trial 1/Trial 2	BR2	12 isolates *G. intermedium* (VPRI 43980), *G. irregulare* (VPRI 43981), *G. sylvaticum* (VPRI 43982), *G. capense* sp. nov. (CBS 149752), *P. lutarium/diclinum* (VPRI 43984), *G. commune* sp. nov. (CBS 149753), *G. recalcitrans* (VPRI 43986), *G. terrestris* (VPRI 43987), *P. tracheiphilum* (UM2130), *P. vanterpoolii* (VPRI 43988), *G. macrosporum* (VPRI 43989), *G. ultimum* var. *ultimum* (VPRI 43979)	5 plates 10 seeds/plate

**Table 6:** Glasshouse post-germination bioassay investigating pathogenicity of *Globisporangium* and *Pythium* spp. on pyrethrum.

Experiment	Trial	Growth Condition	Pyrethrum Seed Cultivars	*Pythium* Isolates	Replication per Treatment
Experiment 1	Trial 1 and Trial 2	Glasshouse at 20–24 °C under natural light	BR2	*G. intermedium* (VPRI 43980); *G. irregulare* (VPRI 43981); *G. sylvaticum* (VPRI 43982); *G. capense* sp. nov. (CBS 149752); *P. lutarium/diclinum* (VPRI 43984); *G. commune* sp. nov. (CBS 149753); *G. recalcitrans* (VPRI 43986); *G. terrestris* (VPRI 43987); *P. tracheiphilum* (UM2130); *G. macrosporum* (VPRI 43989)	12 (Trial 1)/10 (Trial 2)
Experiment 2	Trial 1 and Trial 2	Plant growth chamber at 20–24 °C in a 12/12 light/dark cycle	BR2	*G. ultimum* var. *ultimum* (VPRI 43979)	10

## Data Availability

Data presented in this paper are available from NCBI GenBank database and the Supplementary Materials.

## Supplementary Materials

The following supporting information can be downloaded at: https://www.mdpi.com/article/10.3390/plants12061361/s1, Figure S1: Maximum Likelihood tree with the highest log likelihood (−3064.25) showing phylogenetic relationships among *Pythium*-like species based on *Cox1* regions; Figure S2: Disease symptoms observed in the monitored pyrethrum sites. Table S1: Information relation to the pyrethrum sites surveyed.
