# Supplementary material for: Globisporangium and Pythium Species Associated with Yield Decline of Pyrethrum (Tanacetum cinerariifolium) in Australia
Source: Plants (Basel). 2023 Mar 17;12(6):1361. doi: 10.3390/plants12061361 (PMC10051369; doi:10.3390/plants12061361)
Supplement: Supplementary file 1 [file plants-12-01361-s001.zip › plants-2235428-supplementary.pdf]

Article

Globisporangium and *Pythium* Species Associated with Yield-Decline of Pyrethrum (*Tanacetum cinerariifolium*) in Australia

Yuzhu Liu [1], Niloofar Vaghefi [1], Peter K. Ades [2], Alexander Idnurm [3], Aabroo Ahmed [4] and Paul W. J. Taylor [1,*]

[1] School of Agriculture and Food, Faculty of Science, University of Melbourne, Parkville, VIC 3010, Australia
[2] School of Ecosystem and Forest Sciences, Faculty of Science, University of Melbourne, Parkville, VIC 3010, Australia
[3] School of BioSciences, Faculty of Science, University of Melbourne, Parkville, VIC 3010, Australia
[4] University of Saskatchewan, Canada
* Correspondence: paulwjt@unimelb.edu.au

Supplementary Materials

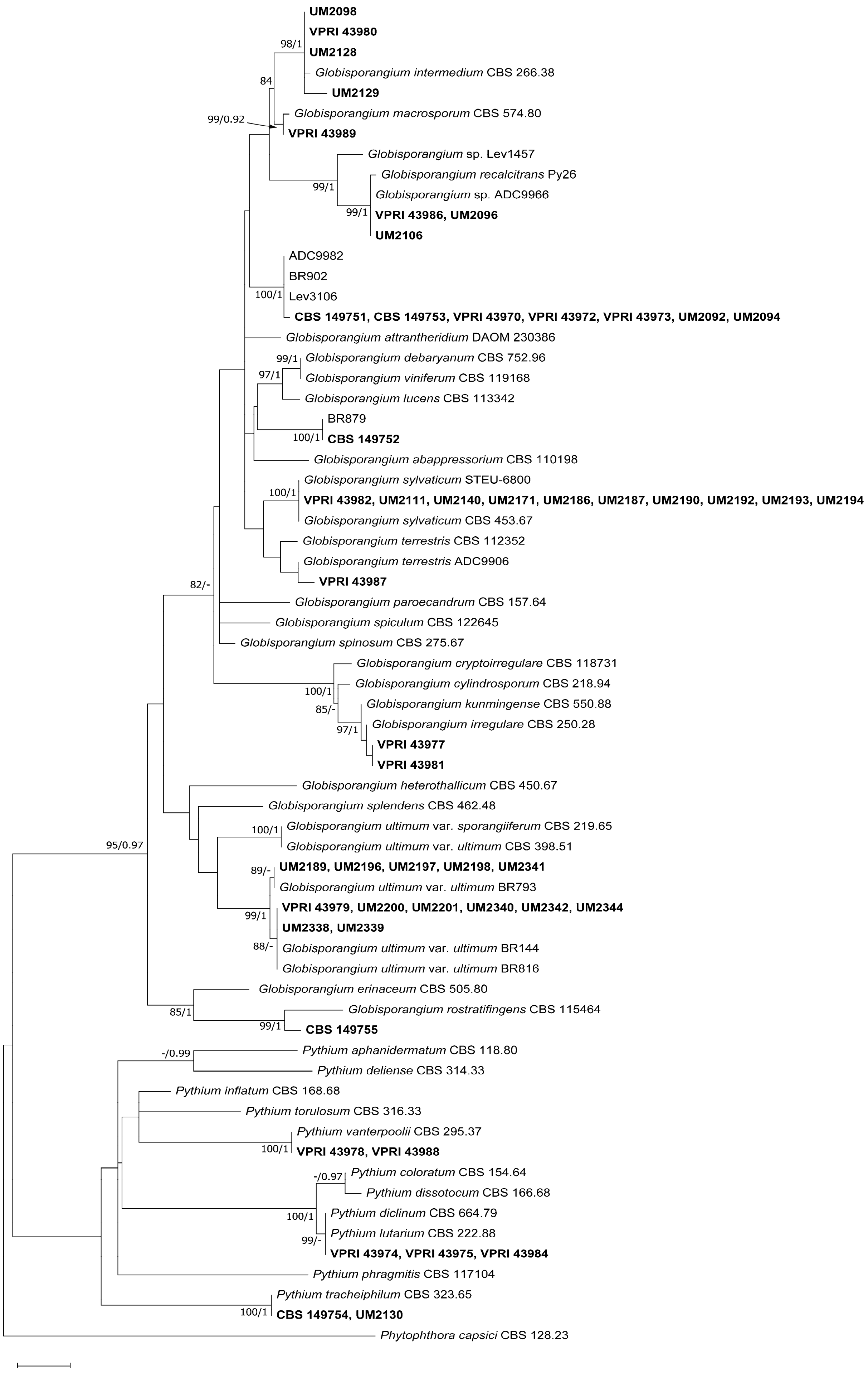
UM2098
VPRI 43980
UM2128
Globisporangium intermedium CBS 266.38
UM2129
98/1
84
99/0.92
Globisporangium macrosporum CBS 574.80
VPRI 43989
Globisporangium sp. Lev1457
Globisporangium recalcitrans Py26
Globisporangium sp. ADC9966
VPRI 43986, UM2096
UM2106
99/1
99/1
ADC9982
BR902
Lev3106
CBS 149751, CBS 149753, VPRI 43970, VPRI 43972, VPRI 43973, UM2092, UM2094
100/1
Globisporangium attrantheridium DAOM 230386
Globisporangium debaryanum CBS 752.96
Globisporangium viniferum CBS 119168
Globisporangium lucens CBS 113342
99/1
97/1
BR879
CBS 149752
100/1
Globisporangium abappressorium CBS 110198
Globisporangium sylvaticum STEU-6800
VPRI 43982, UM2111, UM2140, UM2171, UM2186, UM2187, UM2190, UM2192, UM2193, UM2194
Globisporangium sylvaticum CBS 453.67
100/1
Globisporangium terrestris CBS 112352
Globisporangium terrestris ADC9906
VPRI 43987
82/-
Globisporangium paroecandrum CBS 157.64
Globisporangium spiculum CBS 122645
Globisporangium spinosum CBS 275.67
Globisporangium cryptoirregulare CBS 118731
Globisporangium cylindrosporum CBS 218.94
Globisporangium kunmingense CBS 550.88
Globisporangium irregulare CBS 250.28
VPRI 43977
VPRI 43981
100/1
85/-
97/1
Globisporangium heterothallicum CBS 450.67
Globisporangium splendens CBS 462.48
Globisporangium ultimum var. sporangiiferum CBS 219.65
Globisporangium ultimum var. ultimum CBS 398.51
100/1
UM2189, UM2196, UM2197, UM2198, UM2341
Globisporangium ultimum var. ultimum BR793
VPRI 43979, UM2200, UM2201, UM2340, UM2342, UM2344
UM2338, UM2339
Globisporangium ultimum var. ultimum BR144
Globisporangium ultimum var. ultimum BR816
89/-
99/1
88/-
95/0.97
Globisporangium erinaceum CBS 505.80
Globisporangium rostratifingens CBS 115464
CBS 149755
85/1
99/1
Pythium aphanidermatum CBS 118.80
Pythium deliense CBS 314.33
-/0.99
Pythium inflatum CBS 168.68
Pythium torulosum CBS 316.33
Pythium vanterpoolii CBS 295.37
VPRI 43978, VPRI 43988
100/1
Pythium coloratum CBS 154.64
Pythium dissotocum CBS 166.68
-/0.97
Pythium diclinum CBS 664.79
Pythium lutarium CBS 222.88
VPRI 43974, VPRI 43975, VPRI 43984
100/1
99/-
Pythium phragmitis CBS 117104
Pythium tracheiphilum CBS 323.65
CBS 149754, UM2130
100/1
Phytophthora capsici CBS 128.23

Figure S1. Maximum likelihood tree with the highest log likelihood (-3064.25) showing relationships among *Pythium*-like species based on *Cox1* regions. Isolates included in this study are shown in bold. ML bootstrap support values greater than 75 % and Bayesian posterior probability values greater than 0.90 are indicated at internodes. The scale bar shows the number of nucleotide changes per site. The analysis involved 66 nucleotide sequences. There were a total of 543 positions in the final dataset. The tree is rooted with *Phytophthora capsici* CBS 128.23 [22].

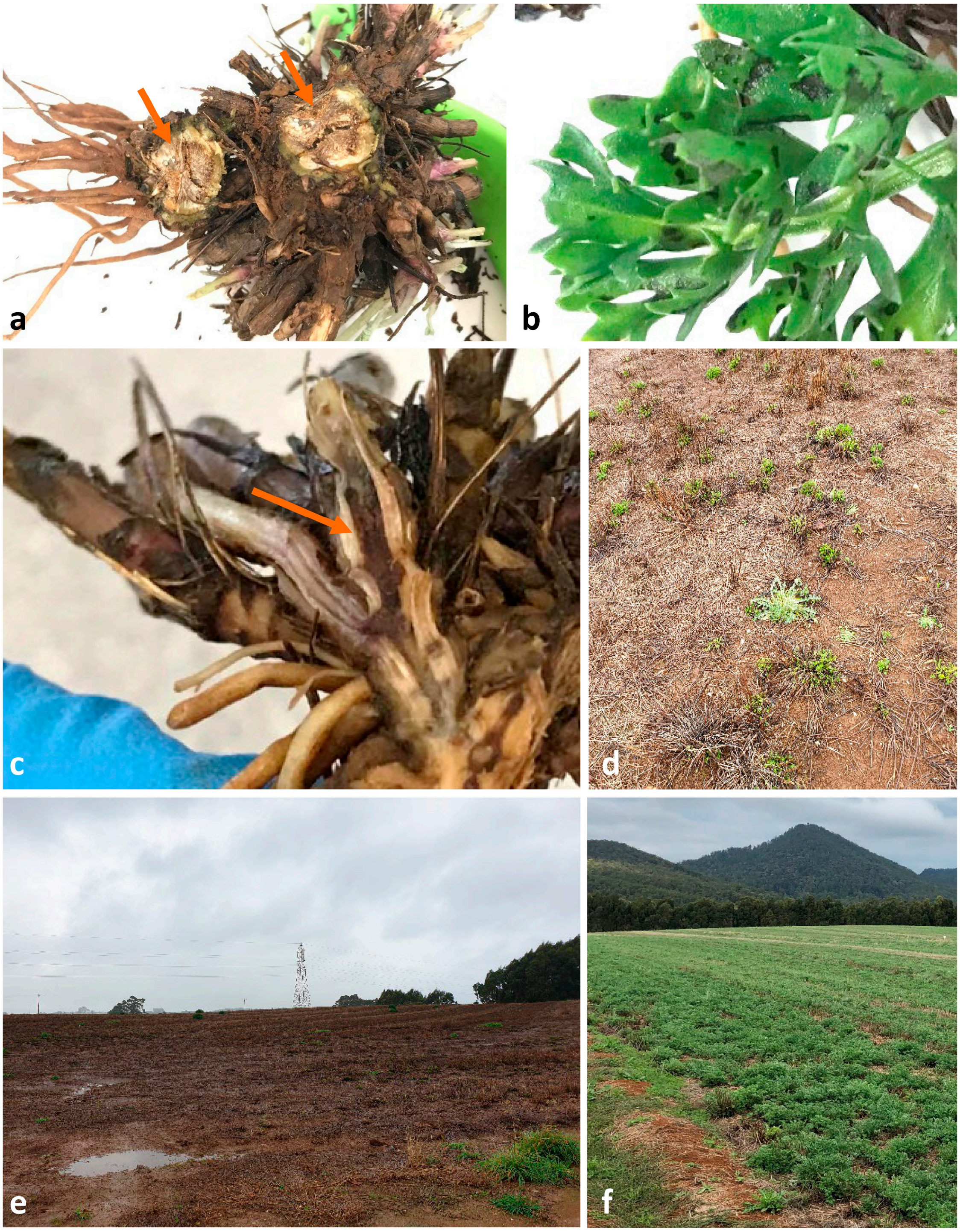

Figure S2. Disease symptoms observed in the monitored pyrethrum sites. **a** brown discoloration of crown tissue (arrows); **b** necrotic leaf lesions; **c** dark brown discoloration of crown tissue (arrow); **d** stunting pyrethrum plant; **e** field with severe poor regrowth in winter; **f** field with good regrowth in autumn.

Table S1. Information related to the pyrethrum sites surveyed.

Site number	Location	Sowing date	Basic information of each site	Sampling date	Plants sampled and comments on site
91808	Dunnstown, VIC	18/8/2016	N/A	06/2018	Three plants showing stunting were selected and sampled.
92012	Bullarook, VIC	29/8/2016	N/A	06/2018	Three plants showing stunting were selected and sampled.
46604	Penguin, TAS	23/08/2018	N/A	12/03/2020	Plants on this site were healthy. Three healthy plants were randomly selected and sampled.
46026	Penguin, TAS	23/08/2018	N/A	12/03/2020	Plants on this site were healthy. Three healthy plants were randomly selected and sampled.
63711	Wynyard, TAS	20/09/2018	N/A	25/06/2020	Severe poor regrowth showing stunting and leaf lesions. Yield of first harvest was 60 kg/ha. Three plants showing stunting and leaf lesions were selected and sampled.
874020	Wynyard, TAS	20/09/2018	N/A	25/06/2020	Plants were healthy with just a few plants starting to show sighs of poor re-growth. Three plants showing stunting and leaf lesions were selected and sampled.
700091	Wynyard, TAS	23/10/2019	N/A	25/06/2020	Site planted on wide row spacing. Plants were a bit small. Three small plants were selected and sampled.
700090	Wynyard, TAS	18/09/2019	N/A	25/06/2020	Site immediately next to 700091. Also, wide row and plants were larger than in 700091. Three plants which are much smaller than the rest were sampled.
70087	Wynyard, TAS	10/10/2018	N/A	25/06/2020	Noticeable poor regrowth showing stunting and leaf lesions. This site was 'terminated' and was not taken through to second harvest. Thus, grower stopped all herbicide treatments in 2020. Three plants showing disease symp-toms were selected and sampled.
646009	Wynyard, TAS	30/09/2019	N/A	25/06/2020	Many small and struggling plants. Three plants showing stunting and leaf le-sions were selected and sampled.
902015	Dean, VIC	08/2020	Red ferrosol soil, herbicide treated, previous crop potatoes	24/12/2020	Seedlings on this site were healthy.
				09/11/2021	Looked uniform and healthy overall with just a few plants showing wilting and stunting. Both asymptomatic and symptomatic plants were sampled.
910013	Dean, VIC	08/2020	Red ferrosol with stone soil, herbicide treated, previous crop canola	24/12/2020	Seedlings on this site were healthy.
				09/11/2021	Looked uniform and healthy overall with just a few plants showing wilting and stunting. Both asymptomatic and symptomatic plants were sampled
918012	Dunnstown, VIC	08/2020	Red ferrosol soil, herbicide treated, previous crop cereal	24/12/2020	Seedlings on this site were healthy.
				09/11/2021	Looked uniform and healthy overall with just a few plants showing wilting and stunting. Both asymptomatic and symptomatic plants were sampled.
922013	Mollongghip, VIC	08/2020	Red ferrosol with stone soil, herbicide treated, previous crop carrots after potatoes. Sample spot is prone to be waterlogged.	24/12/2020	Seedlings on this site were healthy.
				09/11/2021	Looked uniform and healthy overall with just a few plants showing wilting and stunting. Both asymptomatic and symptomatic plants were sampled.
949006	Dunnstown, VIC	08/2020		24/12/2020	Seedlings on this site were healthy. Healthy plants were sampled.

			Red ferrosol soil, herbicide treated, previous crop cereal-wheat	09/11/2021	Looked uniform and healthy. Healthy plants were sampled.
954001	Bullarook, VIC	08/2020	Red ferrosol with stone soil, herbicide treated. Land is owned by water authority and recently leased for farming, only 2019 cropped to potato, first crop of pyrethrum	24/12/2020	Seedlings on this site were healthy. Healthy plants were sampled.
				09/11/2021	Looked uniform and healthy overall with just a few plants showing wilting and stunting. Both asymptomatic and symptomatic plants were sampled.
